# Lupus Autoimmunity and Metabolic Parameters Are Exacerbated Upon High Fat Diet-Induced Obesity Due to TLR7 Signaling

**DOI:** 10.3389/fimmu.2019.02015

**Published:** 2019-09-04

**Authors:** Noël Hanna Kazazian, Yawen Wang, Annie Roussel-Queval, Laetitia Marcadet, Lionel Chasson, Caroline Laprie, Benoit Desnues, Jonathan Charaix, Magali Irla, Lena Alexopoulou

**Affiliations:** Aix Marseille University, CNRS, INSERM, CIML, Marseille, France

**Keywords:** systemic lupus erythematosus (SLE), toll-like receptor 7 (TLR7), metabolic syndrome, obesity, animal model, innate immunity, dendritic cells

## Abstract

Systemic lupus erythematosus (SLE) patients have increased prevalence of metabolic syndrome but the underlying mechanisms are unknown. Toll-like receptor 7 (TLR7) that detects single stranded-RNA plays a key role in antimicrobial host defense and also contributes to the initiation and progression of SLE both in mice and humans. Here, we report the implication of TLR7 signaling in high fat diet (HFD)-induced metabolic syndrome and exacerbation of lupus autoimmunity in TLR8-deficient (TLR8ko) mice, which develop spontaneous lupus-like disease due to increased TLR7 signaling by dendritic cells (DCs). The aggravated SLE pathogenesis in HFD-fed TLR8ko mice was characterized by increased overall immune activation, anti-DNA autoantibody production, and IgG/IgM glomerular deposition that were coupled with increased kidney histopathology. Moreover, upon HFD TLR8ko mice developed metabolic abnormalities, including liver inflammation. In contrast, upon HFD TLR7/8ko mice did not develop SLE and both TLR7ko and TLR7/8ko mice were fully protected from metabolic abnormalities, including body weight gain, insulin resistance, and liver inflammation. Interestingly, HFD led to an increase of TLR7 expression in WT mice, that was coupled with increased TNF production by DCs, and this phenotype was more profound in TLR8ko mice. Our study uncovers the implication of TLR7 signaling in the interconnection of SLE and metabolic abnormalities, indicating that TLR7 might be a novel approach as a tailored therapy in SLE and metabolic diseases.

## Introduction

Epidemiological data provide evidence of a significant increase of autoimmune diseases in the last 30 years and growing attention has focused on the role of environmental factors, especially the western dietary habits that have led to the parallel rise in obesity (1). A link between obesity, metabolic syndrome and autoimmune diseases, including systemic lupus erythematosus (SLE), and the associated chronic inflammation has been suggested, but the underlying mechanisms are still unknown (2, 3).

SLE is a chronic systemic autoimmune disease predominantly affecting young women, during their reproductive years, and is characterized by the presence of autoantibodies against self-nucleic acids and associated proteins (4). Self-nucleic acids released by dying cells are recognized by the autoantibodies and form immune complexes, which accumulate in different organs, leading to increased inflammation and tissue damage. SLE pathogenesis derives from the combination of genetic, environmental, hormonal, and epigenetic factors (4). Current standard of care treatments for SLE are mainly non-selective immunosuppressants. However, not all patients respond to these treatments, and one major side effect of the systemic immunosuppression is the increased incidence of infection (5). Thus, better understanding of the pathogenesis of SLE is pivotal for the development of new treatments.

Metabolic syndrome is characterized by several risk factors that include central obesity, insulin resistance, dyslipidemia, increased blood pressure, and endothelial dysfunction (6). It is highly linked to SLE and is associated with cumulative organ damage (7, 8). Metabolic syndrome is also common in young patients with recently diagnosed SLE. These patients present higher levels of inflammatory markers, than SLE subjects without metabolic syndrome (9). In childhood-onset SLE increased serum tumor necrosis factor (TNF) levels are associated with obesity and body fat content (10). The white adipose tissue is a crucial site for the generation of soluble mediators most of which carry pro-inflammatory activity. These include classical cytokines such as tumor necrosis factor alpha (TNF), interleukin 6 (IL-6), and adipokines such as leptin, adiponectin, and resistin (11). By their pro-inflammatory action, these molecules contribute to low-grade inflammation in obese subjects. Dysfunctional adipose tissue lipid metabolism leads to increased circulating fatty acids that initiates inflammatory signaling cascades in the population of infiltrating cells (11). A feedback loop of pro-inflammatory cytokines exacerbates this pathological state driving further immune infiltration and cytokine secretion and disrupts the insulin signaling cascade. In addition to adipose tissue, other metabolically critical sites, particularly the liver, are also involved during the course of the disease. Fatty liver is characterized by excessive triglyceride deposition as lipid droplets in hepatocytes that trigger mitochondrial oxidative stress and hepatic inflammation with increased TNF, IL-6, and IL-1β, cytokine production, which are considered as critical factors leading to the progression from benign hepatic steatosis to advanced steatohepatitis or even fibrosis and cirrhosis (12). All together, these events lead to what is termed a sterile inflammatory response, which if not appropriately resolved, can lead to the development of chronic inflammation that underlies or exacerbates autoimmunity.

Little is known about the early molecular events that lead to immune cell infiltration and inflammatory cytokine production in adipose tissue or liver. Nevertheless, the Toll-like receptor (TLR) family has recently emerged as a critical link between inflammation and a contributor to obesity and insulin resistance, both in mice and in humans (13–15). Among the 10 human TLR members, TLR2, TLR4, TLR5, and TLR9 have been reported as regulators of metabolic inflammation and insulin resistance in liver and adipose tissue (16–19). TLRs are evolutionary conserved transmembrane receptors that play a critical role in driving innate and adaptive immunity through the recognition of various microbial components and the activation of signaling pathways critical for the induction of inflammatory responses (20). In addition to sensing exogenous microbial ligands, TLRs also detect endogenous molecules released from damaged tissues or dead cells and regulate many sterile inflammatory processes (21). Mammalian endosomal TLRs (TLR3, TLR7, TLR8, and TLR9) and especially TLR7 play an important role in the development of SLE both in humans (22–24) and in mice (25–28). TLR7 and TLR8 are phylogenetically similar and both sense single stranded RNA (ssRNA) in humans, while in the mouse only TLR7 senses ssRNA (29–31). Despite the fact that murine TLR8 does not seem to have a ligand (32, 33), we previously demonstrated that it plays an important biological role by controlling TLR7-mediated lupus. Indeed, TLR8-deficiency in mice on the C57BL/6 background leads to lupus development due to increased TLR7 expression and signaling by DCs (26–28). Hence, tight control and regulation of TLR7 is pivotal for avoiding SLE and inflammatory pathology.

The implication of TLR7 in the chronic inflammation of SLE in the context of obesity or metabolic syndrome has never been reported to date. Given the increased risk of metabolic syndrome in SLE patients and the fact that TLR7 is implicated in SLE development we hypothesized that TLR7 might be the connecting link between metabolic syndrome and SLE. Thus, in the current study we evaluated the impact of high fat diet (HFD) on the development of SLE and metabolic syndrome in TLR8-deficient mice (TLR8ko) that spontaneously develop SLE due to increased TLR7 signaling by DCs (26–28). Our data revealed that in TLR8ko mice HFD exacerbated the development of SLE, and increased the occurrence of metabolic abnormalities. The elevated disease course in HFD-fed TLR8ko mice was accompanied by increased TLR7 expression and signaling in DCs. In contrast to TLR8ko mice, TLR7/8ko mice were fully protected from SLE and metabolic syndrome. Hence, TLR7 signaling is implicated in the connection between SLE and metabolic disease.

## Materials and Methods

### Mice

TLR7ko, TLR8ko, and double TLR7/8ko mice were generated as described previously (26, 31, 34). All three TLR-deficient mouse lines were backcrossed on the C57BL/6 background for more than 10 generations. Familiar transmission shapes the distinct intestinal microbiota of TLR-deficient mice (35). Therefore, in order to normalize the microbiota between the TLR-deficient mice and WT controls and because the TLR7 and TLR8 genes are both located in the X chromosome, female age-matched TLR-deficient mice and their respective WT control mice were derived by mating littermate TLR-heterozygous female mice with WT or TLR-deficient male mice (e.g., TLR8^X+X−^ x TLR8^X+Y^, or TLR8^X−Y^). Mice were allowed to consume water and pellet shew *ad libitum*. For the HFD-induced obesity model, mice received either HFD (Research Diets, D12492; 60% of kcals from fat and 20% of kcals from carbohydrate) or standard diet (SD) (Research Diets, D12450J; 10% of kcals from fat, 70% of kcals from carbohydrate) beginning at 3 months of age. Mice were housed under specific pathogen-free conditions at the Center d'Immunologie de Marseille-Luminy and experiments were conducted in accordance with institutional guidelines for animal care and with protocols approved by the Comité National de Réflexion Ethique sur l'Expérimentation Animale (protocol number 13.325).

### Reagents

R848, LPS from *E. coli* 0111-B4, CpG ODN 1826 and poly I:C were purchased from Invivogen.

### RNA Isolation and Q-PCR

Total RNA was isolated with TRIzol reagent (Ambion, Life Technologies). RNA was reversed transcribed with Superscript II reverse transcriptase (Invitrogen) and Q-PCR for TLR7, TNF, IL-6, IL-1β, IL-10, Foxp3, and β-actin was performed as described previously (26). Primers are listed in Table S1.

### Serological Analysis

Evaluation of IgM, and IgG autoantibodies against DNA and RNA on serum samples were performed as described previously (26).

### Glucose Tolerance Test

Mice fed HFD or SD were injected intraperitonially with D-glucose (1 g/kg body weight) after 6 h fast. Blood was collected from tail tip at the indicated time points and glycemia was determined using a glucometer (ACCU-CHEK, Roche).

### Flow Cytometric Analysis

Mice were euthanized, perfused with 10 ml sterile PBS solution to remove blood cells and then spleen, liver, or adipose tissue were extracted. Spleen was passed through a 200-gauge nylon mesh to obtain a single cell suspension followed by erythrocyte lysis. Splenocytes were digested with digestion solution (RPMI medium containing 2% FCS, 7 mg/ml Collagenase II and 1 mg/ml DNase I) for 20 min at 37°C. Following enzymatic digestion, cell suspension was passed through a 70 μm cell strainer and splenocytes were collected by centrifugation. Isolation of hepatic lymphocytes with mechanical dissection was carried out as follows: liver was cut in small pieces by scissors, suspended in digestion solution, incubated at 37°C for 20 min, cell suspension was passed through a 100 μm cell strainer, centrifuged, and erythrocytes were lysed. After centrifugation the cell pellet was resuspend in 80% Percoll solution, overlaid by a layer of 40% Percoll solution followed by centrifugation at 1,500 g for 20 min, the cells were aspirated from the Percoll interface and harvested by centrifugation. Stromal vascular fraction cells from adipose tissue were isolated with an adipose tissue dissociation kit from Miltenyi Biotec using manufacturer's instructions.

Cell suspensions were incubated with 24G2 hybridoma supernatant and then stained using fluorochrome-labeled antibodies against the following antigens: CD45.2, B220, CD3, NK1.1, CD11b, Ly6G, CD44, CD62L, CD38, CD138, GL7 from BD Biosciences, F4/80, CD4, CD8, IA/IE (MHC class II) from eBioscience and CD11c, CD64, SiglecH, CD69 from Biolegend. For intracellular staining of TLR7 and TNF, cells were fixed with Cytofix (BD Biosciences), permeabilized with 0.1% saponin containing staining buffer and stained in saponin buffer using immunofluorescence labeled antibodies for TLR7 (A94B10 from BD Biosciences) and TNF (MP6-XT22 from BD Biosciences). For intracellular staining of Foxp3, cells were fixed, permeabilized and stained with a Foxp3 staining kit, according to the manufacturer's instructions (FJK-16s from eBioscience). Flow cytometry was conducted using an LSR2 (BD Biosciences) and data were analyzed with FlowJo (Tree Star). The gating strategies for the various cell populations are presented in Figures S1, S2.

### Histology and Immunofluorescence

For histopathology studies, livers were fixed in formalin and embedded in paraffin. For light microscopy 3–4 μm thick tissue sections were stained with hematoxylin and eosin (H&E). To determine the extent of renal and liver damage, biopsies were analyzed by a pathologist. Typical glomerular active lesions of lupus nephritis were evaluated based on glomerular cellularity, glomerular deposits, and interstitial inflammation. At least 20 glomeruli per kidney were evaluated. Kidney scoring was from 0 to 4 corresponding to no, low, moderate, high and severe changes, respectively. Liver scoring was from 0 to 3 [0, no inflammation; 1, mild inflammation (<5% of section area); 2 moderate inflammation (5–10% of section area); and 3 marked inflammation (more than 10% of section area)]. For cryostat sections, livers, and kidneys were embedded in OCT-compound and frozen in liquid nitrogen. Sections were cut on a cryostat at 10 μm, thaw-mounted on gelatinized slides, and immunofluorescence IgG and IgM staining on kidney sections and Oil red O staining on liver sections were performed as described previously (26, 36).

### Statistical Analysis

**Figure 1:**
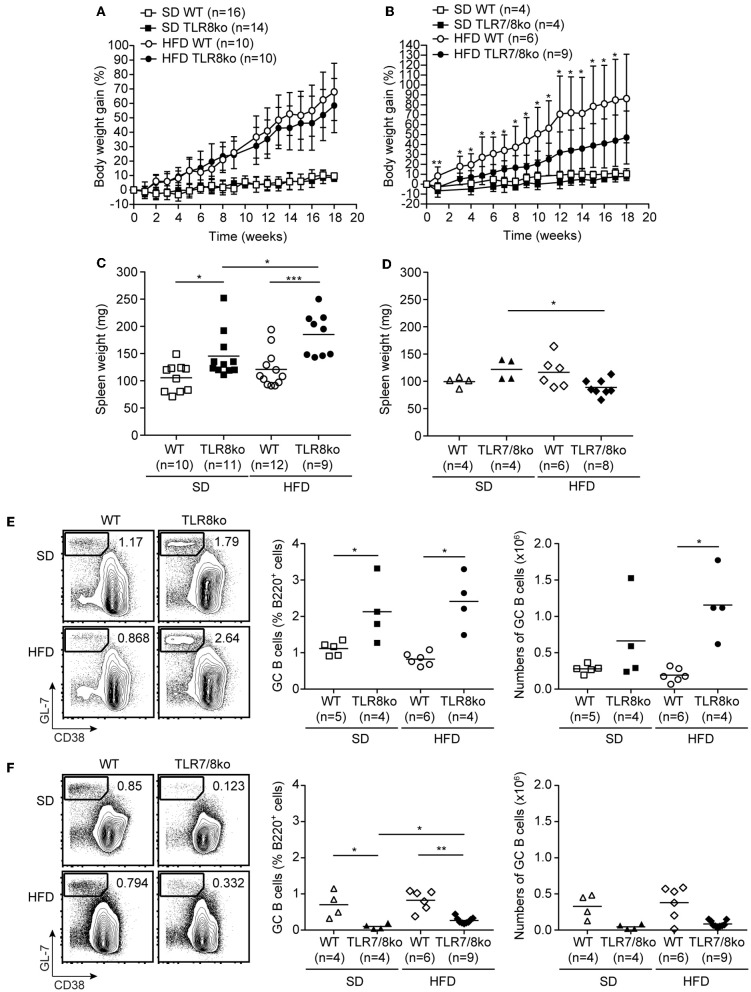
Body weight gain over time of **(A)** TLR8ko or **(B)** TLR7/8ko mice and their respective WT control mice fed a standard diet (SD) or a high fat diet (HFD). Spleen weight of 8 months old female **(C)** TLR8ko or **(D)** TLR7/8ko mice and their WT controls fed-SD or -HFD. Representative flow cytometry plots (left) and graphical analysis (middle and right) of B220^+^GL7^+^CD38^−^ GC B cells in splenocytes derived from 8 months old female **(E)** TLR8ko or **(F)** TLR7/8ko mice and their WT controls fed-SD or -HFD. In **(C,D)** and the right in **(E,F)**, each point represents the value obtained from one mouse and horizontal bars denote mean value. In **(A–D)** data are representative of 2 and 4 independent experiments for SD and HFD, respectively. In **(E,F)** data are representative of 2 independent experiments for each type of diet.

**Figure 2:**
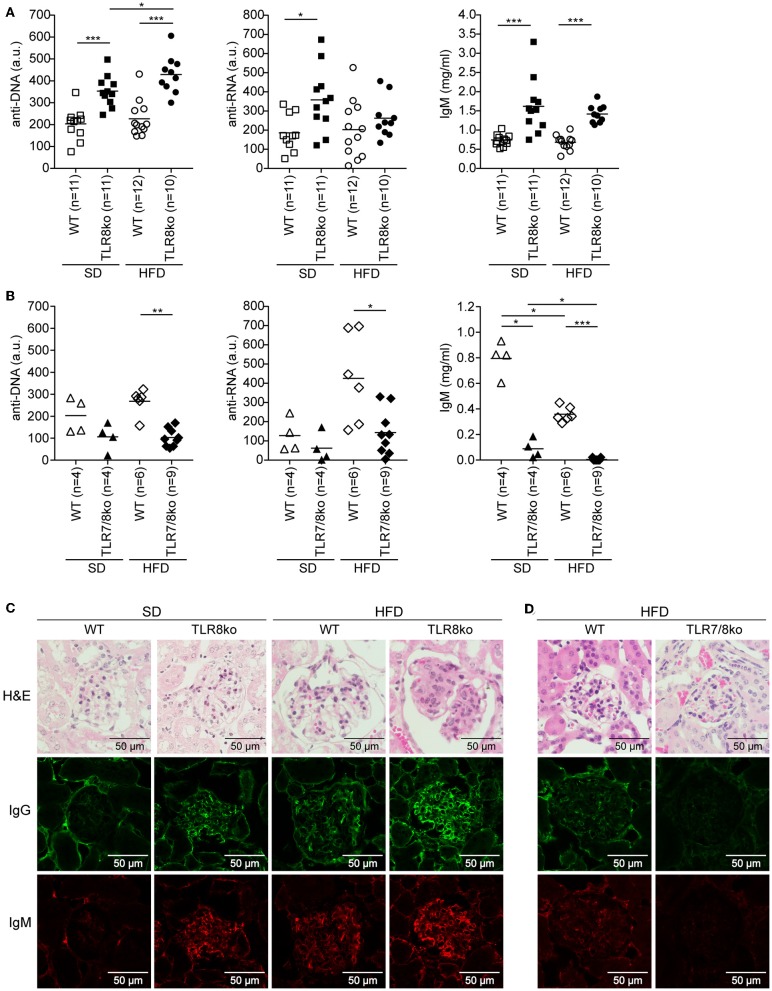
Effect of HFD on serum levels of anti-DNA and anti-RNA autoantibodies and kidney pathology in TLR8ko, TLR7/8ko, and WT mice. Levels of anti-DNA and anti-RNA autoantibodies and IgM in sera of 8 months old female **(A)** TLR8ko or **(B)** TLR7/8ko mice and their WT controls upon SD or HFD were evaluated by ELISA. Each point represents the value of one mouse and horizontal bars denote mean. In **(A)** data are pooled from two independent experiments. Kidney sections from female 8 months old **(C)** SD or HFD-fed TLR8ko and WT or **(D)** HFD-fed TLR7/8ko and WT mice (*n* = 6, for each genotype) were stained with H&E, anti-IgM, or anti-IgG antibodies. In **(A,B)** data are representative of 2 and 4 independent experiments for SD and HFD, respectively. In **(C,D)** data are representative of 2 independent experiments for each type of diet.

**Figure 3:**
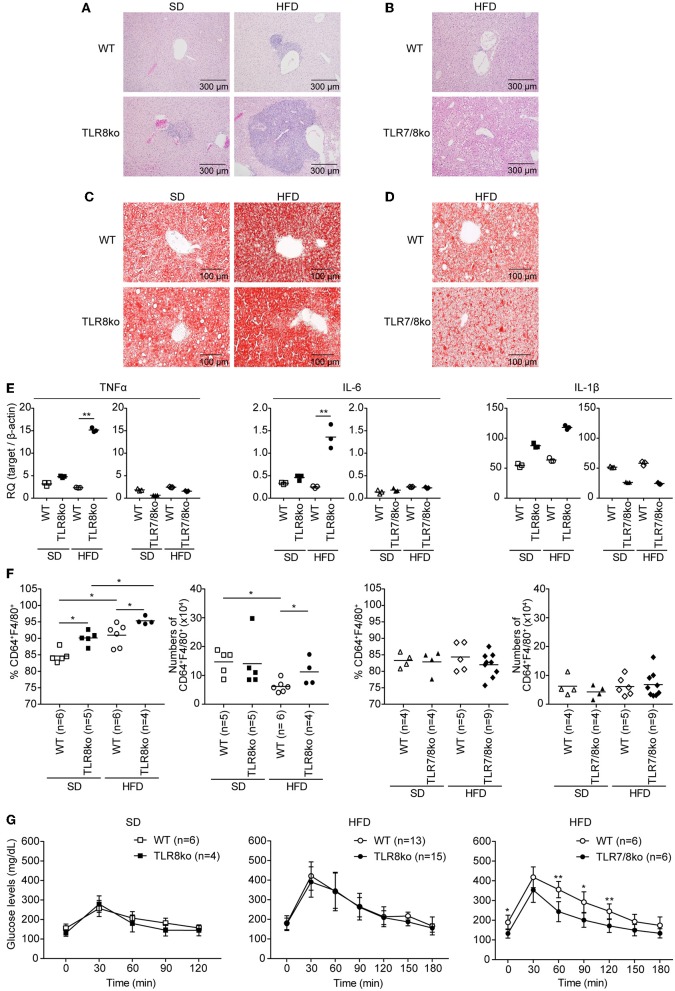
Exacerbation of liver inflammation upon HFD is more profound in TLR8ko vs. WT mice, while TLR7/8ko mice are protected. Liver sections from 8-months-old SD- or HFD-fed TLR8ko or TLR7/8ko mice and their WT controls (*n* = 6 for each genotype) were stained with **(A,B)** H/E or **(C,D)** oil-red O. Data are representative of 2 to 3 independent experiments. **(E)** Expression of TNF, IL-6, and IL-1β mRNA levels was evaluated in the liver of SD- or HFD-fed TLR8ko, TLR7/8ko, or WT mice by Q-PCR. Plots represent mean ± SD of triplicates, with *n* = 4–6 mice combined per genotype. Data are representative of 2 independent experiments. **(F)** Frequency and total numbers of hepatic macrophages (defined as CD45.2^+^MHCII^+^CD11b^+^F4/80^+^CD64^+^) from SD- or HFD-fed TLR8ko, TLR7/8ko, or WT mice were evaluated by FACS analysis. Data are representative of 2 independent experiments. **(G)** Glucose tolerance test (GTT) of 7-months-old SD- or HFD-fed TLR8ko or TLR7/8ko mice and their WT controls. Data are representative of 2–3 independent experiments.

**Figure 4:**
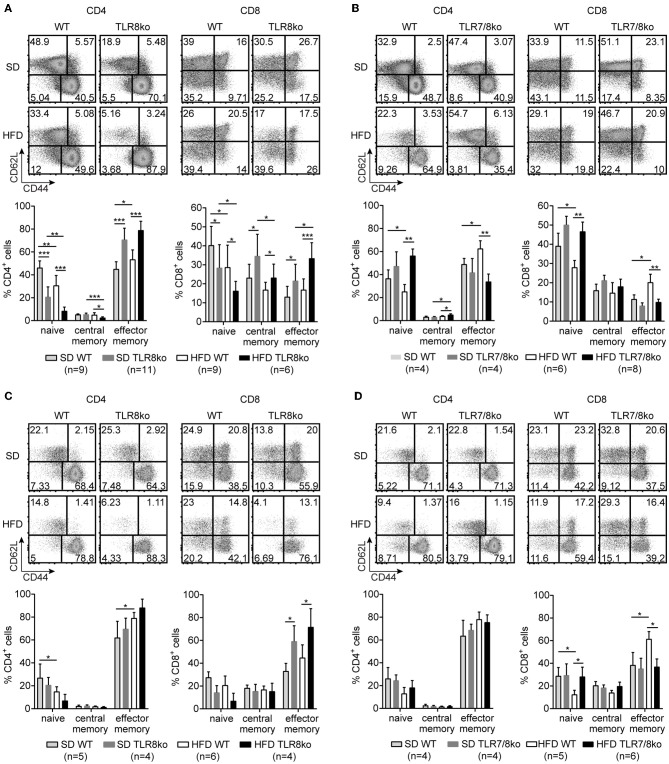
TLR7-dependent increase of effector memory CD4^+^ and CD8^+^ T cells in TLR8ko mice. Representative flow cytometry plots (upper) and graphical analysis (lower) of **(A,B)** splenocytes and **(C,D)** hepatocytes from 8 months old SD- or HFD-fed TLR8ko or TLR7/8ko mice and their WT controls, analyzed for the expression of CD3, CD4, CD8, CD44, and CD62L. CD44/CD62L relative expression were used to identify naive (CD44^lo/−^CD62L^hi^), central memory (CD44^hi^CD62L^hi^), and effector memory (CD44^hi^CD62L^lo^) subpopulations. Plots represent mean ± SD. Data are representative of 2 and 3 independent experiments for SD and HFD, respectively.

**Figure 5:**
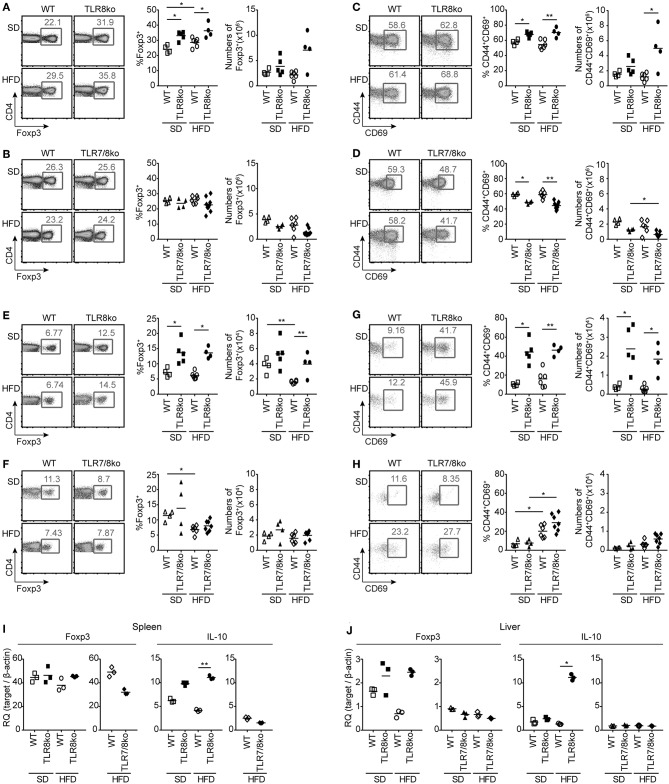
Increased numbers and activation of Treg cells in HFD-fed TLR8ko mice are TLR7-dependent. Total **(A–D)** splenocytes and **(E–H)** hepatic lymphocytes were isolated from 8 months old TLR8ko (SD, *n* = 5; HFD, *n* = 4) and WT (SD, *n* = 4; HFD, *n* = 6) or TLR7/8ko (SD, *n* = 4; HFD, *n* = 7) and WT (SD, *n* = 4; HFD, *n* = 6) mice and analyzed by flow cytometry for **(A,B,E,F)** Treg cells (CD4^+^Foxp3^+^) and **(C,D,G,H)** their activation status (CD44^+^CD69^+^). Expression of Foxp3 and IL-10 mRNA levels were evaluated in the **(I)** spleen and **(J)** liver of SD- or HFD-fed TLR8ko, TLR7/8ko, or WT mice by Q-PCR in triplicates. Plots in **(I–J)** represent mean ± SD of triplicates, with *n* = 4–6 mice combined per genotype. Data in **(A–H)** and **(I,J)** are representative of 3 and 2 independent experiments, respectively.

**Figure 6:**
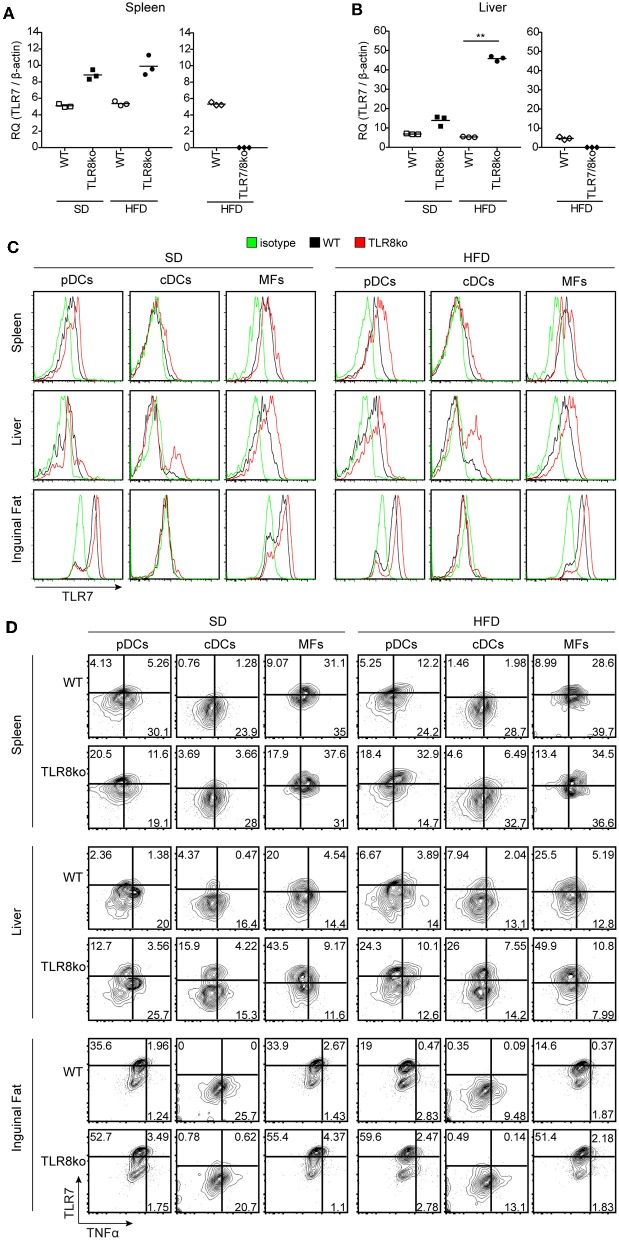
HFD-induced obesity promotes TLR7 and TNF expression by pDCs and cDCs. Total RNA was extracted from **(A)** spleen and **(B)** liver of SD- or HFD-fed WT, TLR8ko, and TLR7/8ko mice and the expression of TLR7 was assessed by Q-PCR. Plots represent mean ± SD of triplicates, with *n* = 4–6 mice combined per genotype. FACS plots represent **(C)** TLR7 expression and **(D)** TLR7 vs. TNF expression on pDCs (CD45.2^+^CD11c^int^SiglecH^+^), cDCs (CD45.2^+^CD11c^+^MHCII^hi^DC64^−^), and macrophages (MFs) (CD45.2^+^CD64^+^CD11c^+^CD11b^+^) derived from the spleen, liver, or inguinal adipose tissue of 8 months-old SD- or HFD-fed female WT and TLR8ko mice (*n* = 3 per genotype). In panel **(D)** gates have been placed relative to isotypes controls as shown in Figure S1. Data in **(A,B)** and **(C,D)** are representative of 2 and 3 independent experiments, respectively.

Statistics were done using Prism 7 (GraphPad Software). In Figures 1A,B, **3G** comparisons were done only between two groups (SD-fed WT vs. TLR8ko mice, or HFD-fed WT vs. TLR8ko mice) and the statistics were done using Wilcoxon rank sum test. Statistics in Figures 1C–F, 2, 3A–F, 4, 5, 6A,B were done by Kruskal-Wallis test followed by Wilcoxon rank sum tests and correction for multiple comparisons using the Benjamini-Hochberg method. *P*-values indicated throughout correspond to: *p* < 0.05 (*), *p* < 0.01 (**), *p* < 0.001 (***), *p* < 0.0001 (****).

## Results

### HFD Aggravates the Lupus Phenotype of TLR8ko Mice

TLR8ko mice on the C57BL/6 background develop lupus due to increased TLR7 expression by DCs (26, 28). To evaluate the effect of high fat diet (HFD) on TLR7-dependent lupus, 3 months old female TLR8ko mice and their respective WT controls (see materials and methods) were fed on either standard diet (SD) or HFD for a total period of 5 months. Total body weight mass was measured weekly after SD or HFD was initiated. HFD-fed mice gained more weight than SD-fed mice, but body weight gain was similar between TLR8ko and WT control mice (Figure 1A). In parallel, we also evaluated the body weight gain of TLR7/8ko and TLR7ko mice vs. their WT controls. Although, upon SD TLR7/8ko mice gained similar weight as their WT controls (Figure 1B), interestingly HFD-fed TLR7/8ko or TLR7ko mice showed reduced weight gain compared to their WT controls (Figure 1B, Figure S3A). The reduced weight gain was not due to differences in food consumption between the genotypes since the assessment of food intake of SD- and HFD-fed TLR8ko, TLR7/8ko, and their WT controls was similar (data not shown). Evaluation of liver, ovarian/uterus, and inguinal white adipose tissue weight, revealed increased fat accumulation in HFD-fed vs. SD-fed mice, but with no evident differences between TLR8ko and WT mice (Figures S4A,B, Table S2), while HFD-fed TLR7/8ko mice had reduced inguinal fat weight than their WT controls (Figures S4C,D, Table S3). These data suggest that upon HFD feeding TLR7-deficiency protects female mice from excess weight gain.

Since TLR8-deficiency in mice spontaneously leads to lupus that is accompanied by splenomegaly and altered splenic immune cell populations (26, 28), we next evaluated these parameters in the context of HFD. SD-fed TLR8ko mice developed splenomegaly compared to SD-fed WT controls, which is in accordance with our previous studies (26, 28), and this phenotype became even more profound in HFD-fed TLR8ko compared to SD-fed TLR8ko mice (Figure 1C, Table S4). On the contrary, SD- and HFD-fed TLR7/8ko and HFD-fed TLR7ko mice had normal spleen weight compared to their WT controls (Figure 1D, Table S5, Figure S3B). Thus, HFD exacerbates splenomegaly in TLR8ko mice and this phenotype is TLR7-dependent since it is abrogated in TLR7/8ko mice.

Flow cytometric analysis of the major immune splenic cell populations (cDCs, pDCs, CD11c^+^ cells, NK, NKT, neutrophils, plasmablasts, B and T cells) revealed no major differences between SD-fed TLR8ko and WT mice (Table S4), except of increased percentages of germinal center (GC) B cells and CD11c^+^ cells in TLR8ko mice (Figure 1E, Table S4), which is in agreement with our previous reports (26, 28). On the contrary, in SD-fed TLR7/8ko mice GC B cells were decreased while the percentages of CD11c^+^ cells were normal, compared to their WT controls (Figure 1F, Table S5). HFD led to an increase of the total numbers of GC B cells in TLR8ko mice, but not in their WT controls (Figure 1E), while in HFD-fed TLR7/8ko or TLR7ko mice this population was similar to WT mice (Figure 1F, Figure S3C). Moreover, the percentages of cDCs were increased in HFD-fed TLR8ko mice, while HFD-fed TLR7/8ko mice had normal percentages (Tables S4, S5). Finally, HFD led to a dramatic reduction of CD8^+^ T cells in TLR8ko, but not in TLR7/8ko mice (Tables S4, S5). Thus, HFD leads to a TLR7-dependent increase of cDCs and reduction of CD8^+^ T cells in TLR8ko mice.

Next, we assessed serum levels of IgG autoantibodies against DNA and RNA, as well as IgM and found increased levels in SD-fed TLR8ko vs. WT mice (Figure 2A). Interestingly, HFD led to an increase of anti-DNA antibodies in HFD-fed TLR8ko vs. SD-fed TLR8ko mice, but that was not the case for anti-RNA antibodies or IgM levels (Figure 2A). In contrast, SD- or HFD-fed TLR7/8ko mice had reduced levels of IgM and IgG autoantibodies against DNA and RNA compared to their WT controls (Figure 2B). These results are in agreement with the differences that we observed in GC B cells in the three genotypes (Figures 1E,F) and strongly suggest that TLR7 is pivotal for autoantibody production.

For renal pathology evaluation, histopathological scoring of glomerular, and interstitial nephritis was performed. HFD-fed TLR8ko mice had increased total kidney and glomerulus score compared to SD-fed TLR8ko or HFD-fed WT mice, while HFD-fed TLR7/8ko mice did not develop any kidney pathology (Figures 2C,D, Figure S5, Table S6). Furthermore, we observed increased IgG and IgM glomerular deposition in HFD-fed TLR8ko mice vs. SD-fed TLR8ko or HFD-fed WT mice (Figure 2C). In contrast, HFD-fed TLR7/8ko and TLR7ko mice showed dramatically diminished glomerular deposition of IgM and IgG compared to HFD-fed WT mice (Figure 2D, Figure S3D). Taken together, these results demonstrate that HFD worsens anti-DNA autoantibody production and glomerular IgM and IgG immunodeposits in TLR8 deficient mice, and that both phenomena depend on TLR7 signaling.

### HFD Exacerbates Liver Inflammation in TLR8ko Mice

Subclinical liver disease is common in SLE (37, 38), thus, in order to determine the effect of HFD and TLR7 expression on liver disease, histopathological evaluation was performed on hematoxylin/eosin and Oil Red O stained liver sections. HFD-fed TLR8ko mice showed an increased average hepatic inflammation with multifocal centrolobular and periportal lymphocytic infiltrates compared to SD-fed TLR8ko and HFD-fed WT mice (Figure 3A, Figure S5, Table S6). Interestingly, HFD-fed TLR7/8ko mice did not develop hepatic inflammation (Figure 3B, Figure S5, Table S6). Moreover, HFD led to a similar increase of the hepatic lipid content, in both TLR8ko and WT mice (Figure 3C), while in HFD-fed TLR7/8ko mice the hepatic lipid content was similar to WT controls (Figure 3D). The increased hepatic inflammation in HFD-fed TLR8ko mice was coupled with increased hepatic mRNA expression of the pro-inflammatory cytokines TNF and IL-6 compared to HFD-fed WT or SD-fed TLR8ko mice (Figure 3E). In contrast, both SD- and HFD-fed TLR7/8ko mice had similar hepatic TNF, IL-6 and IL-1β mRNA levels to their WT controls (Figure 3E). Evaluation by FACS analysis of the major hepatic immune cell populations revealed that HFD-fed TLR8ko mice had increased numbers of CD11c^+^ cells and hepatic macrophages (defined as CD45.2^+^MHCII^+^CD11b^+^F4/80^+^CD64^+^) compared to HFD-fed WT mice (Table S2, Figure 3F), while in HFD-fed TLR7/8ko mice the numbers of CD11c^+^ cells and hepatic macrophages were normal (Table S3, Figure 3F). Evaluation of the activation status of the hepatic macrophages by the expression of CD11c or CD86 did not reveal obvious differences among the three genotypes (data not shown). Therefore, these data demonstrate that HFD consumption leads to a TLR7-dependent hepatic inflammation in lupic TLR8ko mice.

Since liver maintains glucose metabolism, we performed glucose tolerance test (GTT) in SD- and HFD-fed mice. TLR8ko and WT mice showed similar glucose kinetics (Figure 3G), while HFD-fed TLR7/8ko and TLR7ko mice showed improved glucose tolerance compared to their HFD-fed WT controls (Figure 3G, Figure S3E), suggesting that TLR7-deficiency protects from HFD-induced glucose intolerance.

### Increased T Cell Activation and Foxp3^+^ Regulatory T Cells in TLR8ko Mice

Since autoreactive CD4^+^ T cells play an essential role in SLE and steatohepatitis, we next evaluated the percentages/numbers and status of splenic and hepatic T cells. HFD led to a reduction of splenic CD4^+^ and CD8^+^ T cells both in WT and TLR8ko mice compared to SD-fed mice (Table S4), while no differences were observed between SD- and HFD-fed TLR7/8ko mice (Table S5).

The percentages of splenic effector memory (CD44^hi^CD62L^−^) CD4^+^ and CD8^+^ T cells were increased in SD- and HFD-fed TLR8ko vs. WT controls (Figure 4A). On the contrary, the percentages of splenic effector memory CD4^+^ and CD8^+^ T cells were normal in SD-fed TLR7/8ko, whereas HFD induced an increase in WT but not in TLR7/8ko mice (Figure 4B). Accordingly, splenic naïve (CD44^−^CD62L^+^) CD4^+^ and CD8^+^ T cells were reduced in SD-fed TLR8ko vs. WT mice, whereas HFD led to an important reduction of these cell populations in both genotypes (Figure 4A). In contrast, in SD-fed TLR7/8ko mice, the percentages of splenic naïve CD4^+^ and CD8^+^ T cells were similar to WT mice, while HFD led to a reduction in WT, but not in TLR7/8ko mice (Figure 4B).

In the liver, the percentages of effector memory CD4^+^ T cells were similar between TLR8ko, TLR7/8ko and WT mice (Figure 4C). On the contrary, hepatic effector memory CD8^+^ T cells were increased both in SD- and HFD-fed TLR8ko mice compared to their WT controls (Figure 4C). In SD-fed TLR7/8ko mice hepatic effector memory CD8^+^ T cells were similar to WT controls, whereas HFD led to an increase of effector memory CD8^+^ T cells in WT, but not in TLR7/8ko mice (Figure 4D). Respectively, hepatic naïve CD8^+^ T cells percentages were normal in SD-fed TLR7/8ko mice, while HFD induced a strong decrease in their WT controls, but not in HFD-fed TLR7/8ko mice (Figure 4D). Altogether, these data show that both in the spleen and liver HFD leads to an increase of effector memory and decrease of naïve CD4^+^ and CD8^+^ T cells in a TLR7-dependent manner.

CD4^+^ forkhead box protein 3 (Foxp3)^+^ regulatory T (Treg) cells maintain self-tolerance by suppressing autoreactive lymphocytes and are critical regulators of cellular metabolism and glucose homeostasis (39, 40). Considering that defects in Treg cells can contribute in SLE pathogenesis and steatohepatitis (41), we next evaluated splenic and hepatic Treg cells. SD- and HFD-fed TLR8ko mice had increased percentages of Treg cells compared to WT controls, both in the spleen (Figure 5A) and the liver (Figure 5E). The total number of splenic and hepatic Treg cells were similar in TLR8ko and WT mice upon SD (Figures 5A,E), while HFD led to a decrease of hepatic Treg cells in WT mice, but not in TLR8ko mice (Figure 5E). In contrast, TLR7/8ko mice had similar percentages and numbers of splenic and hepatic Treg cells to their WT controls, both upon SD or HFD (Figures 5B,F). In addition, both splenic and hepatic Treg cells where more activated in SD- and HFD-fed TLR8ko mice vs. WT controls (Figures 5C,G). In marked contrast, splenic and hepatic Treg cells of TLR7/8ko mice showed similar activation status to WT cells (Figures 5D,H). No substantial differences were observed regarding splenic or hepatic Foxp3 mRNA between TLR8ko, TLR7/8ko, TLR7ko, and their WT controls (Figures 5I,J, Figure S3F). Given that Treg-mediated suppression can be directly linked to IL-10 production (42), we next assessed splenic and hepatic expression of IL-10. We observed a 2-fold and 5-fold increase of IL-10 mRNA levels in the spleen and liver, respectively, of HFD-fed TLR8ko mice vs. WT controls (Figures 5I,J), while IL-10 levels were normal in TLR7/8ko and TLR7ko mice (Figures 5I,J, Figure S3G). Altogether these results indicate that splenic and hepatic Treg cells are increased in TLR8ko vs. WT mice, and upon HFD this increase is accompanied by elevated levels of the anti-inflammatory cytokine IL-10. Importantly, these phenomena are TLR7-dependent since they are abolished in TLR7/8ko mice.

### HFD Induces a Profound Increase of TLR7^+^TNF^+^ DCs in TLR8ko DCs

To further determine whether exacerbated lupus and metabolic abnormalities observed in HFD- vs. SD-fed TLR8ko mice could be attributed to augmented TLR7 signaling, we next evaluated TLR7 expression levels. While HFD did not have an effect on splenic TLR7 mRNA expression (Figure 6A), it strongly increased of hepatic TLR7 mRNA levels in TLR8ko vs. WT mice (Figure 6B). In contrast, and as expected TLR7 mRNA was undetectable in TLR7/8ko mice (Figures 6A,B). Furthermore, we evaluated TLR7 protein levels by FACS analysis in pDCs, cDCs and macrophages derived from the spleen, liver, and adipose tissue of TLR8ko and WT mice. Upon SD, TLR8ko mice showed higher TLR7 protein levels than WT mice in pDCs, cDCs, and macrophages in all three tissues (Figures 6C,D). Compared to SD-fed mice, HFD led to an increase of TLR7 expression in pDCs and cDCs in both genotypes, but again the HFD-TLR8ko-derived cells had higher TLR7 expression than HFD-WT cells in all three tissues (Figures 6C,D). In contrast, HFD did not affect TLR7 expression in macrophages (Figures 6C,D).

Next, in order to evaluate if the increased TLR7 expression resulted in altered cytokine production, we measured TNF and type I IFN protein levels by flow cytometry. The expression of type I IFN was quite low (data not shown) and difficult to interpret, thus we focused our analysis on TNF production by TLR7 expressing cells. Compared to SD-fed mice, HFD led to an increase of TNF expression mainly in TLR7^+^ pDCs and cDCs derived from the spleen and liver, but not the inguinal fat (Figure 6D). Interestingly, SD-fed TLR8ko mice showed higher TNF expression in TLR7^+^ pDCs and cDCs than SD-fed WT cells, and this difference become even more profound in HFD-fed mice (Figure 6D). However, in macrophages HFD did not increase TNF expression in TLR7^+^ cells either in WT or in TLR8ko mice (Figure 6D). These results are in accordance with our previous findings that TLR8-deficiency in mice leads to increased TLR7 expression and signaling in DCs, but not in macrophages (26, 28). Overall, TLR8ko pDCs and cDCs have higher TLR7 expression than WT cells, whereas HFD-mediated obesity leads to an increase of TLR7^+^TNF^+^ pDCs and cDCs, and this is more profound in TLR8ko than in WT mice.

## Discussion

**Figure 7:**
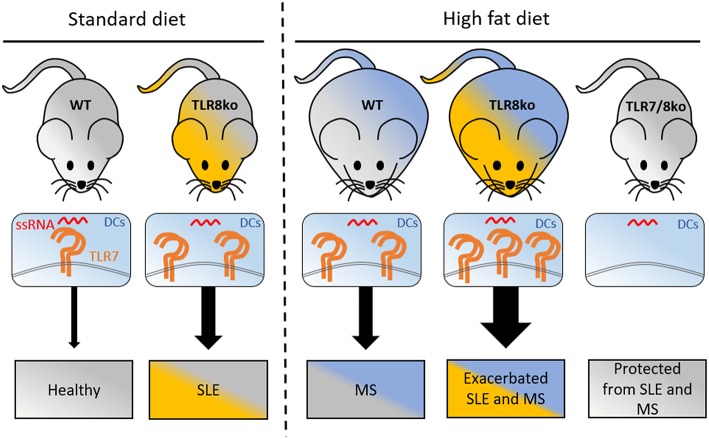
Graphical abstract of how increased TLR7 signaling by DCs can lead to lupus and metabolic syndrome. Under standart diet (SD), TLR8ko mice develop spontaneous SLE due to increased TLR7 expression and signaling by DCs. High fat diet (HFD) leads to the development of MS in WT mice, and exacerbated SLE, and MS in TLR8ko mice, due to increased TLR7 signaling, compared to SD-fed mice. Deletion of TLR7 (TLR7/8ko mice) protects TLR8ko mice from HFD-induced exacerbation of SLE and MS development. DCs, dendritic cells; MS, metabolic syndrome; SLE, systemic lupus erythematosus; ssRNA, single stranded RNA; TLR7, Toll-like receptor 7.

SLE patients have an increased prevalence of metabolic syndrome and this chronic inflammation seems to be associated with cumulative organ damage (7, 8). Mounting evidence suggest an important contribution of TLR7 signaling in lupus disease development both in mice (25–28, 43) and humans (44–47). However, its respective contribution to the metabolic abnormalities observed in the SLE setting is unknown. Using TLR8ko mice on the C57BL/6 background that develop lupus due to increased TLR7 expression and signaling by dendritic cells (26, 28), our data clearly demonstrate that HFD leads to exacerbation of lupus autoimmunity and metabolic parameters and these phenomena could be attributed to increased TLR7 expression and signaling (Figure 7).

Obesity leads to a breakdown of the body's protective self-tolerance, creating the optimal environment for autoimmune diseases, and generates a pro-inflammatory environment likely to worsen disease progression (3). Here, we demonstrated that HFD-induced obesity aggravated the severity of lupus in TLR8ko female mice. This was characterized by profound splenomegaly, kidney IgG and IgM immune-deposits, increased percentages and numbers of GC B cells, anti-DNA serum levels and liver inflammation. B cell-intrinsic TLR7 signaling is essential for the development of germinal centers (48). Indeed, in TLR7/8ko mice the percentages of GC B cells were decreased, while in HFD-fed TLR8ko mice both the percentages and numbers of GC B cells were increased and could explain the increased levels of anti-DNA autoantibodies in HFD-fed TLR8ko mice. Moreover, in accordance with our results previous studies in the female (NZB x NZW) F1 (BWF1) mouse model of SLE have shown that high dietary fat accelerates lupus nephritis and leads to shorter life span (49–51). In HFD-fed TLR8ko mice we did not observe any lethality up to 12 months of age (data not shown), since TLR8ko mice develop a rather mild lupus phenotype compared to BWF1 mice. Indeed, upon SD and up to 12 months TLR8ko mice have normal levels of proteinuria and a 100% survival rate (28), while by the age of 10 months 50% of BWF1 mice develop pathogenic levels of proteinuria and die (51). HFD-fed TLR8ko and WT mice exhibited similar accelerated weight gain, which was coupled with increased inguinal and ovarian fat weight compared to SD-fed control mice. However, female HFD-fed TLR7/8ko and TLR7ko mice showed reduced weight gain and had reduced inguinal fat weight compared to HFD-fed WT controls. This is in agreement with a previous study where upon HFD male TLR7ko mice had normal body weight, but reduced adipose fat pad weight compared to littermate WT mice (52). Strikingly, TLR7/8ko mice did not show any sign of lupus autoimmunity. Thus, TLR7 signaling contributes to the exacerbation of lupus autoimmunity in HFD-fed TLR8ko mice, as well as to the accelerated weight gain that was observed in HFD-fed WT vs. TLR7-deficient mice. Importantly, our study indicates that TLR8ko females provide an ideal mouse model to study the long-term effect of HFD-induced low grade-inflammation in the setting of SLE since mice have a 100% survival rate up to 12 months.

Despite the high heterogeneity in the literature regarding the prevalence of SLE-associated liver diseases, it is becoming apparent that liver is an important target of SLE (53). Subclinical liver disease is common in SLE, where 25–50% of patients with lupus develop abnormal liver function at some point (37, 38). We found that TLR8ko mice developed a TLR7-dependent hepatic inflammation that was accompanied by increased production of pro-inflammatory cytokines (TNF and IL-6), as well as lymphocytic and macrophage infiltrates. Moreover, HFD led to the worsening of the hepatic inflammation in TLR8ko mice. In accordance to our findings regarding the involvement of TLR7 signaling in hepatic inflammation, transgenic mice that overexpress TLR7 develop SLE and massive hepatic inflammation (54), while the contribution of TLR7 signaling in alcoholic hepatic inflammation both in mice and in humans has also been reported recently (55). Therefore, TLR7 signaling contributes to SLE-associated liver diseases.

Treg cells are a subset of CD4^+^ T cells that maintain self-tolerance by suppressing autoreactive lymphocytes. Defects in Treg cells are therefore thought to contribute to SLE pathogenesis (41). Indeed, scurfy mice that are deficient in Treg cells develop generalized autoimmune disorder, including SLE (56). Nevertheless, the data regarding the numbers and function of Treg cells especially in human SLE are contradictory (41). The percentages and numbers of hepatic Treg cells and the activation status of splenic and hepatic Treg cells were increased in TLR8ko mice. These observations correlated with higher hepatic levels of IL-10 in HFD-fed TLR8ko than in HFD-fed WT mice. On the contrary, in TLR7/8ko mice the numbers and activation status of splenic and hepatic Treg cells, as well as hepatic IL-10 levels were normal. These results suggest that TLR7-expression by DCs is pivotal in controlling Treg numbers and their activation status in TLR8ko mice. Interestingly, increased percentages of splenic Treg cells have also been reported in 8 and 16 weeks old female BWF1 mice, which develop spontaneously SLE, hepatic steatosis, and metabolic syndrome (57). However, in the same study it was reported that by the age of 24 weeks the female BWF1 mice had normal percentages of splenic Treg cells, a decline of metabolic symptoms, while classical SLE symptoms increased progressively (57). The observation that Treg cells did not decrease in 8 months old (34 weeks old) TLR8ko mice and even increased upon HFD could be due to the fact that whole body homeostasis deterioration is slower in TLR8ko mice than in BWF1 mice. Indeed, a previous study using BWF1 female mice reported the appearance of lupus-related signs earlier and a 69% survival rate by week 36 (58), while in the study by Vila et al. survival was 100% by week 36 (57). Therefore, the TLR7-dependent increased numbers of Treg cells in TLR8ko mice might have a reparatory role, since Treg cells besides their role in suppressing the immune response are important in tissue protection and regeneration in various tissues (59). Thus, we could hypothesize that the numbers, activation status, heterogeneity, stability, and function of Treg cells can vary during the different stages of SLE progression (60). Furthermore, recent studies have uncovered novel subsets of Treg cells that have a pathogenic role, notably through the secretion of the cytokine IL17A, in the setting of the autoimmune diseases, including SLE and arthritis (61, 62), which could also explain why the role of Treg cells in human SLE remains contradictory. We this could not rule out that Treg cells in HFD-fed TLR8ko mice contain pathogenic cells that contribute to the pathology.

Obesity-related inflammation of metabolic tissues, including liver and adipose tissue, are key elements in the development of metabolic inflammation and insulin resistance, but many of the contributing mechanisms remain ambiguous (63). Our data revealed that in WT mice HFD leads to an increased TLR7 expression that is coupled with increased TNF production especially by splenic and hepatic cDCs and pDCs. Importantly, increased TLR7 expression and signaling was even more profound in TLR8ko mice and correlated with dramatic increase of hepatic cytokines. In accordance with our findings that HFD leads to increased TLR7 expression, a previous study revealed that HFD upregulated the mRNA expression of all TLRs, including TLR7, in the murine adipose tissue, and this phenomenon was coupled by increased NF-κB signaling and cytokine expression (64). Moreover, Revelo et al., showed that HFD-induced obesity in male mice promotes excess release and diminished clearance of nucleic acids, in the form of extracellular traps, that can be detected by TLR7 and can lead to worsening of metabolic inflammation (52). Interestingly, upregulated adipose TLR7 expression and correlation with systemic inflammatory markers has also been reported in obese individuals (65). Thus, we can hypothesize that the increased TLR7 expression and response that we noticed upon HFD mainly in DCs could be the outcome of the activation of DCs either through direct detection of the increased amounts of the endogenous TLR7-ligand and/or increased inflammatory cytokines that lead to DC activation. Increased TLR7 expression coupled with increased TLR7 signaling has detrimental effects on accelerating systemic metabolic inflammation and SLE progression. In line to our findings, calorie restriction in mice delayed the development of kidney disease, age-related immune dysfunction and complications, and thus prolonged the life span in the BWF1 mouse model of lupus (66–68). Information regarding the impact of diet and caloric restriction on human SLE is yet insufficient (2, 69). Nevertheless, caloric restriction was proven to induce various benefits to the immune system (69), since this restriction also leads to changes in the gut microbiota, which act as guardians of a healthy immune system (70).

In conclusion, our data demonstrate that HFD-induced obesity increases TLR7 expression and signaling, that contributes to the acceleration of the progression of SLE and metabolic abnormalities. Since SLE is a systemic inflammatory autoimmune disease with a broad clinical and immunological phenotype, whereas the observed clinical heterogeneity reflects differences in underlying immunopathological process, treatment personalization should be done according to underlying molecular mechanisms (71). It is tempting to speculate that in certain SLE patients the molecular basis of the disease could be due to TLR7 overexpression and signaling, that if it is also coupled with obesity and/or metabolic syndrome this could have detrimental consequences for the initiation, exacerbation and progression of the SLE pathology. Therefore, interventions to reduce or reverse overweight and obesity could improve both symptoms and long-term outcomes of patients with lupus or systemic inflammation, and if this is not achievable then targeting TLR7 is expected to have beneficial effects both for SLE and for metabolic inflammation, especially in certain genetically predisposed individuals.

## Data Availability

All datasets generated for this study are included in the manuscript and/or the Supplementary Files.

## Ethics Statement

Experiments were conducted in accordance with institutional guidelines for animal care and with protocols approved by the Comité National de Réflexion Ethique sur l'Expérimentation Animale.

## Author Contributions

LA and NH designed the experiments and analyzed the data. NH, YW, AR-Q, LM, LC, CL, BD, and JC performed experiments and/or analyzed data. LA supervised the project, and wrote the manuscript with the help of NH and MI.

### Conflict of Interest Statement

The authors declare that the research was conducted in the absence of any commercial or financial relationships that could be construed as a potential conflict of interest.

## Abbreviations

BWF1: (NZB x NZW) F1
DC: dendritic cell
HFD: high fat diet
pDC: plasmacytoid dendritic cell
SD: standard diet
TLR: Toll-like receptor.

## References

[1] Moroni L Bianchi I Lleo A. Geoepidemiology, gender and autoimmune disease. Autoimmun Rev. (2012) 11:A386–92. 10.1016/j.autrev.2011.11.012 22142547

[2] Manzel A Muller DN Hafler DA Erdman SE Linker RA Kleinewietfeld M. Role of Western diet in inflammatory autoimmune diseases. Curr Allergy Asthma Rep. (2014) 14:404. 10.1007/s11882-013-0404-6 24338487 PMC4034518

[3] Versini M Jeandel PY Rosenthal E Shoenfeld Y. Obesity in autoimmune diseases: not a passive bystander. Autoimmun Rev. (2014) 13:981–1000. 10.1016/j.autrev.2014.07.001 25092612

[4] Tsokos GC. Systemic lupus erythematosus. N Engl J Med. (2011) 365:2110–21. 10.1056/NEJMra1100359 22129255

[5] Tektonidou MG Wang Z Dasgupta A Ward MM. Burden of serious infections in adults with systemic lupus erythematosus: a national population-based study, 1996–2011. Arthritis Care Res. (2015) 67:1078–85. 10.1002/acr.22575 25732901 PMC4516647

[6] Deedwania PC Gupta R. Management issues in the metabolic syndrome. J Assoc Physicians India. (2006) 54:797–810. 17214277

[7] Demir S Artim-Esen B Sahinkaya Y Pehlivan O Alpay-Kanitez N Omma A Metabolic syndrome is not only a risk factor for cardiovascular diseases in systemic lupus erythematosus but is also associated with cumulative organ damage: a cross-sectional analysis of 311 patients. Lupus. (2016) 25:177–84. 10.1177/0961203315603140 26354963

[8] Parker B Urowitz MB Gladman DD Lunt M Donn R Bae SC. Impact of early disease factors on metabolic syndrome in systemic lupus erythematosus: data from an international inception cohort. Ann Rheum Dis. (2015) 74:1530–6. 10.1136/annrheumdis-2013-203933 24692585 PMC4515988

[9] Chung CP Avalos I Oeser A Gebretsadik T Shintani A Raggi P. High prevalence of the metabolic syndrome in patients with systemic lupus erythematosus: association with disease characteristics and cardiovascular risk factors. Ann Rheum Dis. (2007) 66:208–14. 10.1136/ard.2006.054973 16901956 PMC1798504

[10] Sinicato NA Postal M Peres FA Pelicari Kde O Marini R dos Santos Ade O. Obesity and cytokines in childhood-onset systemic lupus erythematosus. J Immunol Res. (2014) 2014:162047. 10.1155/2014/162047 24741576 PMC3987792

[11] Hotamisligil GS. Inflammation and metabolic disorders. Nature. (2006) 444:860–7. 10.1038/nature05485 17167474

[12] Cai D Yuan M Frantz DF Melendez PA Hansen L Lee J. Local and systemic insulin resistance resulting from hepatic activation of IKK-beta and NF-kappaB. Nat Med. (2005) 11:183–90. 10.1038/nm1166 15685173 PMC1440292

[13] Fresno M Alvarez R Cuesta N. Toll-like receptors, inflammation, metabolism and obesity. Arch Physiol Biochem. (2011) 117:151–64. 10.3109/13813455.2011.562514 21599616

[14] Konner AC Bruning JC. Toll-like receptors: linking inflammation to metabolism. Trends Endocrinol Metab. (2011) 22:16–23. 10.1016/j.tem.2010.08.007 20888253

[15] Wagner H. Endogenous TLR ligands and autoimmunity. Adv Immunol. (2006) 91:159–73. 10.1016/S0065-2776(06)91004-9 16938540

[16] Garcia-Martinez I Santoro N Chen Y Hoque R Ouyang X Caprio S Hepatocyte mitochondrial DNA drives non-alcoholic steatohepatitis by activation of TLR9. J Clin Invest. (2016) 126:859–64. 10.1172/JCI83885 26808498 PMC4767345

[17] Jialal I Kaur H Devaraj S. Toll-like receptor status in obesity and metabolic syndrome: a translational perspective. J Clin Endocrinol Metab. (2014) 99:39–48. 10.1210/jc.2013-3092 24187406

[18] Jin C Flavell RA. Innate sensors of pathogen and stress: linking inflammation to obesity. J Allergy Clin Immunol. (2013) 132:287–94. 10.1016/j.jaci.2013.06.022 23905917

[19] Nishimoto S Fukuda D Higashikuni Y Tanaka K Hirata Y Murata C. Obesity-induced DNA released from adipocytes stimulates chronic adipose tissue inflammation and insulin resistance. Sci Adv. (2016) 2:e1501332. 10.1126/sciadv.1501332 27051864 PMC4820373

[20] Kawai T Akira S. The role of pattern-recognition receptors in innate immunity: update on Toll-like receptors. Nat Immunol. (2010) 11:373–84. 10.1038/ni.1863 20404851

[21] Lin Q Li M Fang D Fang J Su SB. The essential roles of Toll-like receptor signaling pathways in sterile inflammatory diseases. Int Immunopharmacol. (2011) 11:1422–32. 10.1016/j.intimp.2011.04.026 21600309

[22] Enevold C Nielsen CH Jacobsen RS Hermansen ML Molbo D Avlund K. Single nucleotide polymorphisms in genes encoding toll-like receptors 7, 8, and 9 in Danish patients with systemic lupus erythematosus. Mol Biol Rep. (2014) 41:5755–63. 10.1007/s11033-014-3447-4 24919757

[23] Kawasaki A Furukawa H Kondo Y Ito S Hayashi T Kusaoi M. TLR7 single-nucleotide polymorphisms in the 3' untranslated region and intron 2 independently contribute to systemic lupus erythematosus in Japanese women: a case-control association study. Arthritis Res Ther. (2011) 13:R41. 10.1186/ar3277 21396113 PMC3132023

[24] Shen N Fu Q Deng Y Qian X Zhao J Kaufman KM. Sex-specific association of X-linked Toll-like receptor 7 (TLR7) with male systemic lupus erythematosus. Proc Natl Acad Sci USA. (2010) 107:15838–43. 10.1073/pnas.1001337107 20733074 PMC2936646

[25] Deane JA Pisitkun P Barrett RS Feigenbaum L Town T Ward JM. Control of toll-like receptor 7 expression is essential to restrict autoimmunity and dendritic cell proliferation. Immunity. (2007) 27:801–10. 10.1016/j.immuni.2007.09.009 17997333 PMC2706502

[26] Demaria O Pagni PP Traub S de Gassart A Branzk N Murphy AJ. TLR8 deficiency leads to autoimmunity in mice. J Clin Invest. (2010) 120:3651–62. 10.1172/JCI42081 20811154 PMC2947223

[27] Desnues B Macedo AB Ordonez-Rueda D Roussel-Queval A Malissen B Bruhns P. The transcriptional repressor Gfi1 prevents lupus autoimmunity by restraining TLR7 signaling. Eur J Immunol. (2016) 46:2801–11. 10.1002/eji.201646573 27600904

[28] Desnues B Macedo AB Roussel-Queval A Bonnardel J Henri S Demaria O. TLR8 on dendritic cells and TLR9 on B cells restrain TLR7-mediated spontaneous autoimmunity in C57BL/6 mice. Proc Natl Acad Sci USA. (2014) 111:1497–502. 10.1073/pnas.1314121111 24474776 PMC3910605

[29] Diebold SS Kaisho T Hemmi H Akira S Reis e Sousa C. Innate antiviral responses by means of TLR7-mediated recognition of single-stranded RNA. Science. (2004) 303:1529–31. 10.1126/science.1093616 14976261

[30] Heil F Hemmi H Hochrein H Ampenberger F Kirschning C Akira S. Species-specific recognition of single-stranded RNA via toll-like receptor 7 and 8. Science. (2004) 303:1526–9. 10.1126/science.1093620 14976262

[31] Lund JM Alexopoulou L Sato A Karow M Adams NC Gale NW. Recognition of single-stranded RNA viruses by Toll-like receptor 7. Proc Natl Acad Sci USA. (2004) 101:5598–603. 10.1073/pnas.0400937101 15034168 PMC397437

[32] Alexopoulou L Desnues B Demaria O. [Toll-like receptor 8: the awkward TLR]. Med Sci. (2012) 28:96–102. 10.1051/medsci/2012281023 22289837

[33] Cervantes JL Weinerman B Basole C Salazar JC. TLR8: the forgotten relative revindicated. Cell Mol Immunol. (2012) 9:434–8. 10.1038/cmi.2012.38 23085951 PMC3498840

[34] Valenzuela DM Murphy AJ Frendewey D Gale NW Economides AN Auerbach W. High-throughput engineering of the mouse genome coupled with high-resolution expression analysis. Nat Biotechnol. (2003) 21:652–9. 10.1038/nbt822 12730667

[35] Ubeda C Lipuma L Gobourne A Viale A Leiner I Equinda M. Familial transmission rather than defective innate immunity shapes the distinct intestinal microbiota of TLR-deficient mice. J Exp Med. (2012) 209:1445–56. 10.1084/jem.20120504 22826298 PMC3409501

[36] Traub S Demaria O Chasson L Serra F Desnues B Alexopoulou L. Sex bias in susceptibility to MCMV infection: implication of TLR9. PLoS ONE. (2012) 7:e45171. 10.1371/journal.pone.0045171 23028824 PMC3447886

[37] Luangjaru S Kullavanijaya P. Gastrointestinal and hepatobiliary manifestations in systemic lupus erythematosus. J Med Assoc Thai. (2005) 88:71–5. 15960221

[38] van Hoek B. The spectrum of liver disease in systemic lupus erythematosus. Neth J Med. (1996) 48:244–53. 10.1016/0300-2977(96)00003-4 8710047

[39] Josefowicz SZ Lu LF Rudensky AY. Regulatory T cells: mechanisms of differentiation and function. Annu Rev Immunol. (2012) 30:531–64. 10.1146/annurev.immunol.25.022106.141623 22224781 PMC6066374

[40] Matarese G Procaccini C De Rosa V Horvath TL La Cava A. Regulatory T cells in obesity: the leptin connection. Trends Mol Med. (2010) 16:247–56. 10.1016/j.molmed.2010.04.002 20493774

[41] Ohl K Tenbrock K. Regulatory T cells in systemic lupus erythematosus. Eur J Immunol. (2015) 45:344–55. 10.1002/eji.201344280 25378177

[42] Chaudhry A Samstein RM Treuting P Liang Y Pils MC Heinrich JM. Interleukin-10 signaling in regulatory T cells is required for suppression of Th17 cell-mediated inflammation. Immunity. (2011) 34:566–78. 10.1016/j.immuni.2011.03.018 21511185 PMC3088485

[43] Pisitkun P Deane JA Difilippantonio MJ Tarasenko T Satterthwaite AB Bolland S. Autoreactive B cell responses to RNA-related antigens due to TLR7 gene duplication. Science. (2006) 312:1669–72. 10.1126/science.1124978 16709748

[44] Jenks SA Cashman KS Zumaquero E Marigorta UM Patel AV Wang X. Distinct effector B cells induced by unregulated Toll-like receptor 7 contribute to pathogenic responses in systemic lupus erythematosus. Immunity. (2018) 49:725–39.e6. 10.1016/j.immuni.2018.08.015 30314758 PMC6217820

[45] Lee YH Choi SJ Ji JD Song GG. Association between toll-like receptor polymorphisms and systemic lupus erythematosus: a meta-analysis update. Lupus. (2016) 25:593–601. 10.1177/0961203315622823 26762473

[46] Murayama G Furusawa N Chiba A Yamaji K Tamura N Miyake S. Enhanced IFN-alpha production is associated with increased TLR7 retention in the lysosomes of palasmacytoid dendritic cells in systemic lupus erythematosus. Arthritis Res Ther. (2017) 19:234. 10.1186/s13075-017-1441-7 29052537 PMC5649081

[47] Souyris M Mejia JE Chaumeil J Guery JC. Female predisposition to TLR7-driven autoimmunity: gene dosage and the escape from X chromosome inactivation. Semin Immunopathol. (2018) 41:153–64. 10.1007/s00281-018-0712-y 30276444

[48] Soni C Wong EB Domeier PP Khan TN Satoh T Akira S. B cell-intrinsic TLR7 signaling is essential for the development of spontaneous germinal centers. J Immunol. (2014) 193:4400–14. 10.4049/jimmunol.1401720 25252960 PMC4201954

[49] Alexander NJ Smythe NL Jokinen MP. The type of dietary fat affects the severity of autoimmune disease in NZB/NZW mice. Am J Pathol. (1987) 127:106–21. 3565532 PMC1899609

[50] Gilbert EL Ryan MJ. High dietary fat promotes visceral obesity and impaired endothelial function in female mice with systemic lupus erythematosus. Gend Med. (2011) 8:150–5. 10.1016/j.genm.2011.03.006 21536233 PMC3229028

[51] Kelley VE Izui S. Enriched lipid diet accelerates lupus nephritis in NZB x W mice. Synergistic action of immune complexes and lipid in glomerular injury. Am J Pathol. (1983) 111:288–97. 6344647 PMC1916288

[52] Revelo XS Ghazarian M Chng MH Luck H Kim JH Zeng K. Nucleic acid-targeting pathways promote inflammation in obesity-related insulin resistance. Cell Rep. (2016) 16:717–30. 10.1016/j.celrep.2016.06.024 27373163 PMC6354586

[53] Bessone F Poles N Roma MG. Challenge of liver disease in systemic lupus erythematosus: clues for diagnosis and hints for pathogenesis. World J Hepatol. (2014) 6:394–409. 10.4254/wjh.v6.i6.394 25018850 PMC4081614

[54] Sun X Wiedeman A Agrawal N Teal TH Tanaka L Hudkins KL. Increased ribonuclease expression reduces inflammation and prolongs survival in TLR7 transgenic mice. J Immunol. (2013) 190:2536–43. 10.4049/jimmunol.1202689 23382559 PMC3594466

[55] Massey VL Qin L Cabezas J Caballeria J Sancho-Bru P Bataller R. TLR7-let-7 signaling contributes to ethanol-induced hepatic inflammatory response in mice and in alcoholic hepatitis. Alcohol Clin Exp Res. (2018) 42:2107–22. 10.1111/acer.13871 30103265 PMC6282707

[56] Hadaschik EN Wei X Leiss H Heckmann B Niederreiter B Steiner G. Regulatory T cell-deficient scurfy mice develop systemic autoimmune features resembling lupus-like disease. Arthritis Res Ther. (2015) 17:35. 10.1186/s13075-015-0538-0 25890083 PMC4391674

[57] Vila L Roglans N Baena M Barroso E Alegret M Merlos M. Metabolic alterations and increased liver mTOR expression precede the development of autoimmune disease in a murine model of lupus erythematosus. PLoS ONE. (2012) 7:e51118. 10.1371/journal.pone.0051118 23226562 PMC3514194

[58] Alperovich G Rama I Lloberas N Franquesa M Poveda R Goma M. New immunosuppresor strategies in the treatment of murine lupus nephritis. Lupus. (2007) 16:18–24. 10.1177/0961203306073136 17283580

[59] Li J Tan J Martino MM Lui KO. Regulatory T-cells: potential regulator of tissue repair and regeneration. Front Immunol. (2018) 9:585. 10.3389/fimmu.2018.00585 29662491 PMC5890151

[60] Overacre AE Vignali DA. T(reg) stability: to be or not to be. Curr Opin Immunol. (2016) 39:39–43. 10.1016/j.coi.2015.12.009 26774863 PMC4801724

[61] Kluger MA Nosko A Ramcke T Goerke B Meyer MC Wegscheid C. RORgammat expression in Tregs promotes systemic lupus erythematosus via IL-17 secretion, alteration of Treg phenotype and suppression of Th2 responses. Clin Exp Immunol. (2017) 188:63–78. 10.1111/cei.12905 27880975 PMC5343349

[62] Komatsu N Okamoto K Sawa S Nakashima T Oh-hora M, Kodama T, et al. Pathogenic conversion of Foxp3+ T cells into TH17 cells in autoimmune arthritis. Nat Med. (2014) 20:62–8. 10.1038/nm.3432 24362934

[63] Osborn O Olefsky JM. The cellular and signaling networks linking the immune system and metabolism in disease. Nat Med. (2012) 18:363–74. 10.1038/nm.2627 22395709

[64] Kim SJ Choi Y Choi YH Park T. Obesity activates toll-like receptor-mediated proinflammatory signaling cascades in the adipose tissue of mice. J Nutr Biochem. (2012) 23:113–22. 10.1016/j.jnutbio.2010.10.012 21414767

[65] Sindhu S Wilson A Akhter N Shenouda S Kochumon S Behbehani K Increased adipose tissue expression of Toll-like receptor (TLR)-7 in obese individuals: significance in metabolic disease. J Glycom Lipidom. (2015) 5:1000136 10.4172/2153-0637.1000136

[66] Fernandes G Yunis EJ Good RA. Influence of diet on survival of mice. Proc Natl Acad Sci USA. (1976) 73:1279–83. 10.1073/pnas.73.4.1279 1063408 PMC430247

[67] Jolly CA Muthukumar A Avula CP Troyer D Fernandes G. Life span is prolonged in food-restricted autoimmune-prone (NZB x NZW)F(1) mice fed a diet enriched with (n-3) fatty acids. J Nutr. (2001) 131:2753–60. 10.1093/jn/131.10.2753 11584100

[68] Muthukumar AR Jolly CA Zaman K Fernandes G. Calorie restriction decreases proinflammatory cytokines and polymeric Ig receptor expression in the submandibular glands of autoimmune prone (NZB x NZW)F1 mice. J Clin Immunol. (2000) 20:354–61. 10.1023/A:1006620130114 11051277

[69] Constantin MM Nita IE Olteanu R Constantin T Bucur S Matei C. Significance and impact of dietary factors on systemic lupus erythematosus pathogenesis. Exp Ther Med. (2019) 17:1085–90. 10.3892/etm.2018.6986 30679978 PMC6327661

[70] Rosser EC Mauri C. A clinical update on the significance of the gut microbiota in systemic autoimmunity. J Autoimmun. (2016) 74:85–93. 10.1016/j.jaut.2016.06.009 27481556

[71] Banchereau R Hong S Cantarel B Baldwin N Baisch J Edens M Personalized immunomonitoring uncovers molecular networks that stratify lupus patients. Cell. (2016) 165:551–65. 10.1016/j.cell.2016.03.008 27040498 PMC5426482

