# Supplementary material for: Lupus Autoimmunity and Metabolic Parameters Are Exacerbated Upon High Fat Diet-Induced Obesity Due to TLR7 Signaling
Source: Front Immunol. 2019 Sep 4;10:2015. doi: 10.3389/fimmu.2019.02015 (PMC6738575; doi:10.3389/fimmu.2019.02015)
Supplement: Supplementary file 1 [file Table_1.docx]

| Gene | Forward (5’🡪3’) | Reverse (5’🡪3’) |
| --- | --- | --- |
| Tlr7 | TGGCTCCCTTCTCAGGATGA | CCGTGTCCACATCGAAAACA |
| Tnf | TTCTATGGCCCAGACCCTCA | CAGCTGCTCCTCCACTTGGT |
| IL-6 | TGTTCTCTGGGAAATCGTGGA | TTTCTGCAAGTGCATCATCGTT |
| IL-1β | CAAAAGATGAAGGGCTGCTT | GAAGCTGGATGCTCTCATCA |
| IL-10 | CTCTTCACCTGCTCCACTGC | GAATTCCCTGGGTGAGAAGC |
| Foxp3 | CCCACCTACAGGCCCTTCTC | GGCATGGGCATCCACAGT |
| β-actin | TGGAATCCTGTGGCATCCATGAAACC | TAAAACGCAGCTCAGTAACAGTCCG |

**Table S1.** Primers used for Q-PCR
