# Supplementary material for: Lupus Autoimmunity and Metabolic Parameters Are Exacerbated Upon High Fat Diet-Induced Obesity Due to TLR7 Signaling
Source: Front Immunol. 2019 Sep 4;10:2015. doi: 10.3389/fimmu.2019.02015 (PMC6738575; doi:10.3389/fimmu.2019.02015)
Supplement: Supplementary file 2 [file Table_2.docx]

**Table S2.** Liver weight, total cell counts and major cell populations of WT and TLR8ko mice upon SD or HFD.

| Type of diet | | Standard diet (SD) | | High fat diet (HFD) | |
| --- | --- | --- | --- | --- | --- |
| Genotype | | WT  (n=6) | TLR8ko (n=6) | WT  (n=6) | TLR8ko (n=4) |
| Body weight (g) | | 26.6 ± 1.1 | 24.2 ± 1.8 | 35.1 ± 3.2 | 30.9 ± 2.3 |
| Liver weight (g) | | 1.2 ± 0.3 | 0.9 ± 0.1 | 1.1 ± 0.1 | 1.1 ± 0.1‡ |
| Total cell count (x10^4^) |  | 195.3 ± 76 | 134.4 ± 40.7 | 126.3 ± 20 | 166.8 ± 51.4 |
| Cell type | Surface markers |  |  |  |  |
| CD3^+^ cells | CD3^+^ | 45.8 ± 3.4 | 47 ± 4.5 | 46 ± 3.5 | 46.5 ± 5.5 |
|  |  | (88.9 ± 34.4) | (63.3 ± 21) | (58 ± 9.5) | (77.7 ± 24.5) |
| CD4 T cells | CD3^+^CD4^+^ | 19.5 ± 1.6 | 20.1 ± 2.9 | 18.3 ± 1.7 | 14.4 ± 4.3 |
|  |  | (37.3 ± 12.7) | (27.7 ± 10.8) | (23 ± 4.1) | (23.8 ± 9.1) |
| CD8 T cells | CD3^+^CD8^+^ | 15.5 ± 3.5 | 15.1 ± 3.1 | 10.3 ± 2 | 13.8 ± 4.4 |
|  |  | (30.4 ± 13.2) | (21 ± 6.5) | (13 ± 2.8†) | (22.6 ± 8.7) |
| NK cells | NK1.1^+^CD3^-^ | 6.4 ± 0.7 | 6 ± 1.8 | 7.1 ± 0.6 | 7 ± 1.9 |
|  |  | (12.4 ± 5) | (6.8 ± 2) | (9 ± 1.4) | (12.1 ± 6.1) |
| B cells | CD45.2^+^B220^+^ | 23.2 ± 3.5 | 30.4 ± 7.6 | 28.4 ± 7.4 | 20.3 ± 7.8 |
|  |  | (45.1 ± 18.2) | (43.9 ± 18) | (35.8 ± 10.7) | (31.5 ± 10.2) |
| CD11c^+^ cells | CD45.2^+^CD11c^+^ | 14.4 ± 1.1 | 17.5 ± 5.9 | 15.4 ± 3 | 30.9 ± 12.3*‡ |
|  |  | (28.1 ± 10.9) | (20.5 ± 7.3) | (19.8 ± 6.3) | (54.1 ± 36.1*‡) |
| cDC | CD45.2^+^CD11c^hi^MHCII^hi^ CD64^-^ | 1.3 ± 0.1 | 1.5 ± 1 | 1.7 ± 0.4 | 1.4 ± 0.2 |
|  |  | (2.5 ± 0.9) | (1.5 ± 0.6) | (2.1 ± 0.6) | (2.4 ± 1) |
| pDC | CD45.2^+^B220^+^SiglecH^+^ | 1.3 ± 0.6 | 1.1 ± 0.6 | 1 ± 0.3 | 1 ± 0.5 |
|  |  | (2.5 ± 1.6) | (1.3 ± 0.7) | (1.3 ± 0.5) | (1.8 ± 1.4) |
| Neutrophils | CD45.2^+^Ly6G^+^CD11b^+^ | 1.2 ± 0.6 | 1.1 ± 0.9 | 0.9 ± 0.5 | 0.9 ± 0.5 |
|  |  | (2.5 ± 1.5) | (1.5 ± 1.4) | (1.1 ± 0.6) | (1.5 ± 1) |

Data are from 8 months old female mice (n=4-7 per group). Values correspond to the percentage on live lymphocytes for T and NK cells and on live cells for the rest of the cells and shown as average ± SD. In parenthesis, absolute number of cells (x10^4^) are indicated. Data of TLR8ko mice and their WT controls upon SD or HFD are representative of two independent experiments. Statistical analysis was done using Kruskal-Wallis test followed by Mann-Whitney tests. P-values were corrected with the Benjamini and Hochberg method. * P < 0.05 versus corresponding WT, † P < 0.05 versus WT upon SD, ‡ P < 0.05 versus TLR8ko upon SD.
