# Supplementary material for: Lupus Autoimmunity and Metabolic Parameters Are Exacerbated Upon High Fat Diet-Induced Obesity Due to TLR7 Signaling
Source: Front Immunol. 2019 Sep 4;10:2015. doi: 10.3389/fimmu.2019.02015 (PMC6738575; doi:10.3389/fimmu.2019.02015)
Supplement: Supplementary file 3 [file Table_3.docx]

**Table S3.** Liver weight, total cell count, and major cell populations of WT and TLR7/8ko mice upon SD or HFD.

| Type of diet | | Standard diet (SD) | | High fat diet (HFD) | |
| --- | --- | --- | --- | --- | --- |
| Genotype | | WT  (n=4) | TLR7/8ko (n=4) | WT  (n=6) | TLR7/8ko (n=6) |
| Body weight (g) | | 23.9 ± 1.9 | 23.9 ± 2.2 | 38.8 ± 9.5 | 30.4 ± 8.6 |
| Liver weight (g) | | 1 ± 0.2 | 1 ± 0.2 | 1.2 ± 0.3 | 0.9 ± 0.2 |
| Total cell count (x10^4^) |  | 112.1 ± 38.7 | 106.8 ± 41.6 | 136.3 ± 39 | 115.2 ± 29.2 |
| Cell type | Surface markers |  |  |  |  |
| CD3^+^ cells | CD3^+^ | 48.1 ± 2 | 53.5 ± 4.3 | 56 ± 3.7† | 60.6 ± 2.7‡ |
|  |  | (53.8 ± 17.8) | (55.8 ± 16.6) | (77 ± 25.9) | (69.7 ± 17.5) |
| CD4 T cells | CD3^+^CD4^+^ | 17 ± 2.4 | 19.3 ± 1.4 | 17.4 ± 5.3 | 22.7 ± 3.8 |
|  |  | (18.9 ± 6.2) | (20.4 ± 7.2) | (25.1 ± 15.1) | (26.2 ± 7.9) |
| CD8 T cells | CD3^+^CD8^+^ | 14.9 ± 4.3 | 17.1 ± 3.6 | 14.7 ± 2.7 | 14.6 ± 2.8 |
|  |  | (17.2 ± 7.6) | (17.3 ± 3.5) | (20.1 ± 6.7) | (16.8 ± 5) |
| NK cells | NK1.1^+^CD3^-^ | 8.2 ± 3.1 | 7.8 ± 3.8 | 6.3 ± 1.9 | 7.8 ± 2.6 |
|  |  | (8.8 ± 4.1) | (9.4 ± 8.6) | (8.3 ± 2.8) | (9.5 ± 5.1) |
| B cells | CD45.2^+^B220^+^ | 35.2 ± 6.2 | 31.2 ± 3.5 | 31.1 ± 6.9 | 23.2 ± 3.3 |
|  |  | (41 ± 18.4) | (34 ± 15.9) | (42.6 ± 15.1) | (26.5 ± 6.8) |
| CD11c^+^ cells | CD45.2^+^CD11c^+^ | 17.6 ± 7.1 | 17.4 ± 2.9 | 16 ± 4.7 | 16.5 ± 2.2 |
|  |  | (18.3 ± 6.2) | (19.4 ± 10.4) | (21.3 ± 7.6) | (19.3 ± 6.9) |
| cDC | CD45.2^+^CD11c^hi^MHCII^hi^ CD64^-^ | 1.7 ± 1.5 | 1.1 ± 0.2 | 1.1 ± 0.4 | 1.4 ± 0.3 |
|  |  | (1.6 ± 0.7) | (1.2 ± 0.7) | (1.4 ± 0.4) | (1.5 ± 0.3) |
| pDC | CD45.2^+^B220^+^SiglecH^+^ | 1.1 ± 0.6 | 1.8 ± 0.6 | 1 ± 0.7 | 2.7 ± 0.9 |
|  |  | (1.1 ± 0.3) | (2 ± 1) | (1.3 ± 0.7) | (3.2 ± 1.5) |
| Neutrophils | CD45.2^+^Ly6G^+^CD11b^+^ | 2.9 ± 1.3 | 3.5 ± 1.5 | 1.9 ± 0.6 | 1.9 ± 0.7 |
|  |  | (3.5 ± 2.6) | (4.1 ± 3.2) | (2.5 ± 1.1) | (2.2 ± 1.1) |

Data are from 8 months old female mice (n=4-6 per group). Values correspond to the percentage on live lymphocytes for T and NK cells and on live cells for the rest of the cells and shown as average ± SD. In parenthesis, absolute number of cells (x10^4^) are indicated. Data of TLR7/8ko mice and their WT controls upon SD or HFD are representative of two independent experiments. Statistical analysis was done using Kruskal-Wallis test followed by Mann-Whitney tests. P-values were corrected with the Benjamini and Hochberg method. * P < 0.05 versus corresponding WT, † P < 0.05 versus WT upon SD, ‡ P < 0.05 versus TLR7/8ko upon SD.
