# Supplementary material for: Lupus Autoimmunity and Metabolic Parameters Are Exacerbated Upon High Fat Diet-Induced Obesity Due to TLR7 Signaling
Source: Front Immunol. 2019 Sep 4;10:2015. doi: 10.3389/fimmu.2019.02015 (PMC6738575; doi:10.3389/fimmu.2019.02015)
Supplement: Supplementary file 4 [file Table_4.docx]

**Table S4**. Spleen weight, total cell count, and major cell populations of WT and TLR8ko mice upon SD or HFD.

| Type of diet | | Standard diet (SD) | | High fat diet (HFD) | |
| --- | --- | --- | --- | --- | --- |
| Genotype | | WT  (n=4) | TLR8ko (n=5) | WT  (n=6) | TLR8ko  (n=4) |
| Body weight (g) | | 20.1 ± 0.8 | 20.5 ± 0.9 | 35.1 ± 3.2 | 30.9 ± 2.3 |
| Spleen weight (mg) | | 78.5 ± 5.2 | 134.4 ± 17.2* | 100.7 ± 14.6† | 184.3 ± 49.6*‡ |
| Total cell count (x10^6^) |  | 57.5 ± 5 | 61.9 ± 34.4 | 53.7 ± 6.7 | 93.1 ± 39.8 |
| Cell type | Surface markers |  |  |  |  |
| T cells | CD3^+^ | 29.9 ± 2.8 | 28.1 ± 1.6 | 20.1 ± 2.7† | 19.1 ± 2.6‡ |
|  |  | (17.3 ± 2.9) | (17.3 ± 9.4) | (10.6 ± 1.9†) | (18.2 ± 8.6) |
| CD4 T cells | CD3^+^CD4^+^ | 17.3 ± 1.6 | 16.7 ± 1.1 | 12.1 ± 2† | 13.4 ± 1.8‡ |
|  |  | (10 ± 1.7) | (10.1 ± 4.8) | (6.5 ± 1.4) | (13 ± 6.2) |
| CD8 T cells | CD3^+^CD8^+^ | 9.9 ± 1.4 | 8 ± 1.7 | 6.1 ± 1.3† | 3.6 ± 1.7*‡ |
|  |  | (5.7 ± 1.2) | (5.1 ± 3.3) | (3.1 ± 0.6†) | (3.3 ± 2.3) |
| NK cells | NK1.1^+^CD3^-^ | 2.4 ± 0.3 | 2.8 ± 0.3 | 2.8 ± 0.4 | 2.7 ± 0.3 |
|  |  | (1.4 ± 0.2) | (1.7 ± 1) | (1.5 ± 0.2) | (2.4 ± 0.9) |
| NKT cells | NK1.1^+^CD3^+^ | 1.2 ± 0.1 | 1.5 ± 0.2* | 0.9 ± 0.2 | 1 ± 0.3‡ |
|  |  | (0.7 ± 0.1) | (1 ± 0.6) | (0.5 ± 0.11) | (1 ± 0.6) |
| B cells | CD45.2^+^B220^+^ | 58.1 ± 1.9 | 58.1 ± 0.5 | 64 ± 2† | 57 ± 3.8* |
|  |  | (33.3 ± 2.1) | (36 ± 20) | (34.5 ± 4.4) | (53.8 ± 23.9) |
| Plasmablasts | CD45.2^+^B220^-^CD138^+^ | 0.2 ± 0.04 | 0.2 ± 0.1 | 0.2 ± 0.05 | 0.2 ± 0.1 |
|  |  | (0.1 ± 0.02) | (0.1 ± 0.06) | (0.1 ± 0.01) | (0.2 ± 0.1) |
| CD11c^+^ cells | CD45.2^+^CD11c^+^ | 6 ± 0.3 | 10.9 ± 2* | 6.2 ± 1.6 | 13 ± 2.9* |
|  |  | (3.5 ± 0.5) | (6.3 ± 2.5*) | (3.4 ± 1.2) | (12.3 ± 6.8*) |
| cDC | CD45.2^+^CD11c^hi^MHCII^hi^CD64^-^ | 0.9 ± 0.1 | 1 ± 0.1 | 0.9 ± 0.2 | 1.7 ± 0.5*‡ |
|  |  | (0.5 ± 0.1) | (0.6 ± 0.3) | (0.5 ± 0.2) | (1.5 ± 0.6*‡) |
| pDC | CD45.2^+^ B220^+^SiglecH^+^ | 0.4 ± 0.1 | 0.4 ± 0.2 | 0.3 ± 0.05 | 0.4 ± 0.1 |
|  |  | (0.2 ± 0.1) | (0.3 ± 0.28) | (0.2 ± 0.03) | (0.4 ± 0.3) |
| Neutrophils | CD45.2^+^ Ly6G^+^CD11b^+^ | 1.5 ± 0.8 | 2 ± 0.6 | 1.7 ± 0.5 | 2.8 ± 1 |
|  |  | (0.9 ± 0.4) | (1.4 ± 1.1) | (0.9 ± 0.2) | (2.4 ± 0.9*) |

Data are from 8 months old female mice (n=4-6 per group). Values correspond to the percentage on live lymphocytes for T, NK and NKT cells and on live cells for the rest of the cells and shown as average ± SD. In parenthesis, absolute number of cells (x10^6^) are indicated. Data of TLR8ko mice and their WT controls upon SD or HFD are representative of two independent experiments. Statistical analysis was done using Kruskal-Wallis test followed by Mann-Whitney tests. P-values were corrected with the Benjamini and Hochberg method. * P < 0.05 versus corresponding WT, † P < 0.05 versus WT upon SD, ‡ P < 0.05 versus TLR8ko upon SD.
