# Supplementary material for: Lupus Autoimmunity and Metabolic Parameters Are Exacerbated Upon High Fat Diet-Induced Obesity Due to TLR7 Signaling
Source: Front Immunol. 2019 Sep 4;10:2015. doi: 10.3389/fimmu.2019.02015 (PMC6738575; doi:10.3389/fimmu.2019.02015)
Supplement: Supplementary file 5 [file Table_5.docx]

**Table S5.** Spleen weight, total cell count, and major cell populations of WT and TLR7/8ko mice upon SD or HFD.

| Type of diet | | Standard diet (SD) | | High fat diet (HFD) | |
| --- | --- | --- | --- | --- | --- |
| Genotype | | WT  (n=4) | TLR7/8ko (n=4) | WT  (n=6) | TLR7/8lko (n=6) |
| Body weight (g) | | 23.9 ± 1.9 | 23.9 ± 2.2 | 38.8 ± 9.5 | 30.4 ± 8.6 |
| Spleen weight (mg) | | 99.5 ± 9.1 | 121.8 ± 19.1 | 116.7 ± 28.4 | 82.8 ± 10.8*‡ |
| Total cell count (x10^6^) |  | 83.9 ± 12 | 81.2 ± 18 | 69.9 ± 36.7 | 38.4 ± 8.9‡ |
| Cell type | Surface markers |  |  |  |  |
| T cells | CD3^+^ | 25.7 ± 1.4 | 22 ± 1.5 | 22.1 ± 3.5 | 22.3 ± 1 |
|  |  | (21.6 ± 3.8) | (17.8 ± 3.9) | (15 ± 8.3) | (9.3 ± 2‡) |
| CD4 T cells | CD3^+^CD4^+^ | 14.5 ± 1.4 | 10.4 ± 1.5 | 13.3 ± 1.8 | 10.4 ± 0.4* |
|  |  | (12.1 ± 2.2) | (8.3 ± 1.5) | (9.4 ± 5.2) | (4.4 ± 1‡) |
| CD8 T cells | CD3^+^CD8^+^ | 8.3 ± 0.6 | 9.2 ± 0.7 | 6.4 ± 1.7 | 9.5 ± 1 |
|  |  | (7 ± 1.3) | (7.5 ± 2.1) | (4.2 ± 2.4) | (4 ± 1) |
| NK cells | NK1.1^+^CD3^-^ | 2.5 ± 0.7 | 2.8 ± 0.6 | 2 ± 0.3 | 2.4 ± 0.4 |
|  |  | (2.1 ± 0.5) | (2.3 ± 0.8) | (1.3 ± 0.6) | (1 ± 0.1‡) |
| NKT cells | NK1.1^+^CD3^+^ | 1.2 ± 0.2 | 0.8 ± 0.1 | 1.3 ± 0.3 | 1.2 ± 0.3‡ |
|  |  | (1 ± 0.3) | (0.7 ± 0.2) | (0.9 ± 0.5) | (0.5 ± 0.1) |
| B cells | CD45.2^+^B220^+^ | 65.2 ± 0.2 | 65.6 ± 2.2 | 67.5 ± 2.4 | 69.1 ± 1.4‡ |
|  |  | (54.7 ± 7.9) | (53.2 ± 12.2) | (47.6 ± 25.1) | (29 ± 6.4) |
| Plasmablasts | CD45.2^+^ B220^-^CD138^+^ | 0.2 ± 0.03 | 0.1 ± 0.04 | 0.2 ± 0.02 | 0.1 ± 0.01* |
|  |  | (0.1 ± 0.02) | (0.1 ± 0.01) | (0.1 ± 0.06) | (0.05 ± 0.01‡) |
| CD11c^+^ cells | CD45.2^+^CD11c^+^ | 7.9 ± 1.2 | 7.4 ± 1 | 7.7 ± 3.2 | 5.4 ± 0.4*‡ |
|  |  | (6.5 ± 0.3) | (5.9 ± 1.2) | (4.6 ± 2.3) | (2.2 ± 0.4‡) |
| cDC | CD45.2^+^CD11c^hi^MHCII^hi^CD64^-^ | 0.9 ± 0.2 | 0.8 ± 0.1 | 1.1 ± 0.5 | 0.9 ± 0.1 |
|  |  | (0.7 ± 0.1) | (0.7 ± 0.1) | (0.6 ± 0.3) | (0.4 ± 0.09) |
| pDC | CD45.2^+^ B220^+^SiglecH^+^ | 0.4 ± 0.1 | 0.6 ± 0.2 | 0.4 ± 0.1 | 0.6 ± 0.1 |
|  |  | (0.3 ± 0.1) | (0.5 ± 0.2) | (0.3 ± 0.2) | (0.2 ± 0.06) |
| Neutrophils | CD45.2^+^ Ly6G^+^CD11b^+^ | 1.1 ± 0.7 | 2.7 ± 0.9 | 1 ± 0.2 | 1.1 ± 0.2‡ |
|  |  | (0.9 ± 0.5) | (2.2 ± 1) | (0.7 ± 0.4) | (0.5 ± 0.12‡) |

Data are from 8 months old female mice (n=4-6 per group). Values correspond to the percentage on live lymphocytes for T, NK and NKT cells and on live cells for the rest of the cells and shown as average ± SD. In parenthesis, absolute number of cells (x10^6^) are indicated. Data of TLR7/8ko mice and their WT controls upon SD or HFD are representative of two independent experiments. Statistical analysis was done using Kruskal-Wallis test followed by Mann-Whitney tests. P-values were corrected with the Benjamini and Hochberg method. * P < 0.05 versus corresponding WT littermates, † P < 0.05 versus WT upon SD, ‡ P < 0.05 versus TLR7/8ko upon SD.
