# Supplementary material for: Lupus Autoimmunity and Metabolic Parameters Are Exacerbated Upon High Fat Diet-Induced Obesity Due to TLR7 Signaling
Source: Front Immunol. 2019 Sep 4;10:2015. doi: 10.3389/fimmu.2019.02015 (PMC6738575; doi:10.3389/fimmu.2019.02015)
Supplement: Supplementary file 6 [file Table_6.docx]

**Table S6.** Renal and liver histological scores of WT, TLR8ko and TLR7/8ko female 8 months old mice upon SD or HFD

| Diet and genotype | Glomerular cellularity | Glomerular deposits | Total Glomerular score | Interstitial infiltration score | Total kidney score | Liver score |
| --- | --- | --- | --- | --- | --- | --- |
| SD WT | 0.3 ± 0.5 | 0.5 ± 0.8 | 0.11 ± 0.19 | 0.3 ± 0.5 | 0.4 ± 0.5 | 0.1 ± 0.3 |
| SD TLR8ko | 3.5 ± 3.1 | 1.0 ± 1.2 | 0.21 ± 0.20 | 0.8 ± 0.4 | 1.0 ± 0.5 | 0.4 ± 0.5 |
| HFD WT | 2.0 ± 0.9 | 1.8 ± 1.1 | 0. 18 ± 0.09 | 0.3 ±0.5 | 0.5 ± 0.4 | 0.3 ± 0.4 |
| HFD TLR8ko | 13.5 ± 5.9 | 10.6 ± 6.7 | 1.20 ± 0.58 | 0.8 ± 0.4 | 2.0 ± 0.4 | 0.8 ± 0.6 |
| HFD WT | 6.6 ± 2.3 | 2.3 ± 1.7 | 0.44 ± 0.10 | 0.3 ± 0.5 | 0.8 ± 0.5 | 0.1 ± 0.4 |
| HFD TLR7/8ko | 1.1 ± 1.7 | 1.1 ± 2.1 | 0.11 ± 0.19 | 0.0 ± 0.0 | 0.1 ± 0.2 | 0.0 ± 0.0 |

Histopathological scoring of H&E stained paraffin embedded kidney and liver sections of WT, TLR8ko and TLR7/8ko female 8 months old mice upon SD or HFD are displayed as mean ± SD. Pathological scoring method and definitions can be found in Materials and Methods. For kidney histological scoring n=5-6 mice per group. For liver histological scoring n=11-13 mice per group for SD-fed and HFD-fed WT and TLR8ko mice; and for HFD-fed WT and TLR7/8ko mice, n=5 and n=6 mice per group, respectively.
