# Supplementary material for: Lupus Autoimmunity and Metabolic Parameters Are Exacerbated Upon High Fat Diet-Induced Obesity Due to TLR7 Signaling
Source: Front Immunol. 2019 Sep 4;10:2015. doi: 10.3389/fimmu.2019.02015 (PMC6738575; doi:10.3389/fimmu.2019.02015)
Supplement: Supplementary file 7 [file Data_Sheet_1.docx]

**SUPPLEMENTARY FIGURE LEGENDS**

**Figure S1**. Establishment of the gating for TNF expression in TLR7^+^ cells in pDCs (CD45.2^+^CD11c^int^SiglecH^+^), cDCs (CD45.2^+^CD11c^+^MHCII^hi^CD64^-^) and macrophages (MF) (CD45.2^+^CD64^+^CD11c^+^CD11b^+^) isolated from the spleen, liver and inguinal adipose tissue of WT mice. Cells were stained with isotype controls for TLR7 and TNF.

**Figure S2**. Gating strategies used to identify cell populations in the different tissues analysed. Cells from spleen (A) or liver (B) were gated via FSC/SSC profiles, and debris and doublets were excluded. Dead cells were excluded using sytox dye staining. In the spleen, GC B cells were determined as B220^+^ GL7^+^CD38^-^ (fraction a). Spleen and liver cells were stained for CD3, then separated into CD8 T cells (fraction b) and CD4^+^ T cells (fraction c), and further divided into naïve (CD62L^hi^CD44^lo/-^ fraction d), central memory (CD62L^hi^CD44^hi^, fraction e) and effector memory (CD62L^lo^CD44^hi^, fraction f) subpopulations. Treg cells were identified in the spleen and liver as CD4^+^Foxp3^+^ cells (fraction g), among which activated Treg cells were determined as CD44^+^CD69^+^ (fraction h). (C) Cells from liver were gated on live cells and debris and doublets were excluded. Macrophages were gated on CD45.2^+^ hematopoietic cells and determined as CD11b^+^MHCII^+^ F4/80^+^CD64^+^ (fraction i).

**Figure S3.**

Phenotype of HFD-fed female TLR7ko mice versus their WT controls. (A) Body weight gain over time of HFD-fed TLR7ko mice and their respective WT controls. (B) Spleen weight of HFD-fed 8 months-old TLR7ko mice and their WT controls. (C) Percentages of splenic GC B cells (B220^+^GL7^+^CD38^-^) of 8 months-old TLR7ko (n=5) mice and WT controls (n = 4). (D) Kidney sections from 8 months-old TLR7ko and WT mice were stained with anti-IgM or anti-IgG. (E) Glucose tolerance test (GTT) of 7-months-old HFD-fed TLR7ko and their WT controls. Expression of Foxp3 and IL-10 mRNA levels were evaluated in the (F) spleen and (G) liver of HFD-fed TLR7ko or WT mice by Q-PCR. Plots represent mean ± SD of TLR7ko (n = 5) and WT (n = 4) mice. *p<0.05, **p<0.01 and ****p<0.0001 were determined with Mann-Whitney U test.

**Figure S4**.

Inguinal and uterine/ovarian weights of SD- and HFD-fed TLR8ko, TLR7/8ko and WT control mice. (A and C) Inguinal and (B and D) uterine/ovarian fat weight of 8 months-old (A and B) TLR8ko and WT controls or (C and D) TLR7/8ko and WT controls upon SD or HFD. Data are representative of at least 2 independent experiments. *p<0.05, **p<0.01 and ***p<0.001 were determined with Kruskal-Wallis test followed by Mann-Whitney tests and correction for multiple comparisons using the Benjamini and Hochberg method.

**Figure S5.**

Graphical representation of the histological scoring data presented in Table S6 on kidney and liver sections of WT, TLR8ko mice and TLR7/8ko mice female 8 months old upon SD or HFD. Data are representative of at least 2 independent experiments. *p<0.05 and **p<0.01were determined with Kruskal-Wallis test followed by Mann-Whitney tests and correction for multiple comparisons using the Benjamini and Hochberg method.
