# Supplementary figures and images for: Lupus Autoimmunity and Metabolic Parameters Are Exacerbated Upon High Fat Diet-Induced Obesity Due to TLR7 Signaling
Source: Front Immunol. 2019 Sep 4;10:2015. doi: 10.3389/fimmu.2019.02015 (PMC6738575; doi:10.3389/fimmu.2019.02015)

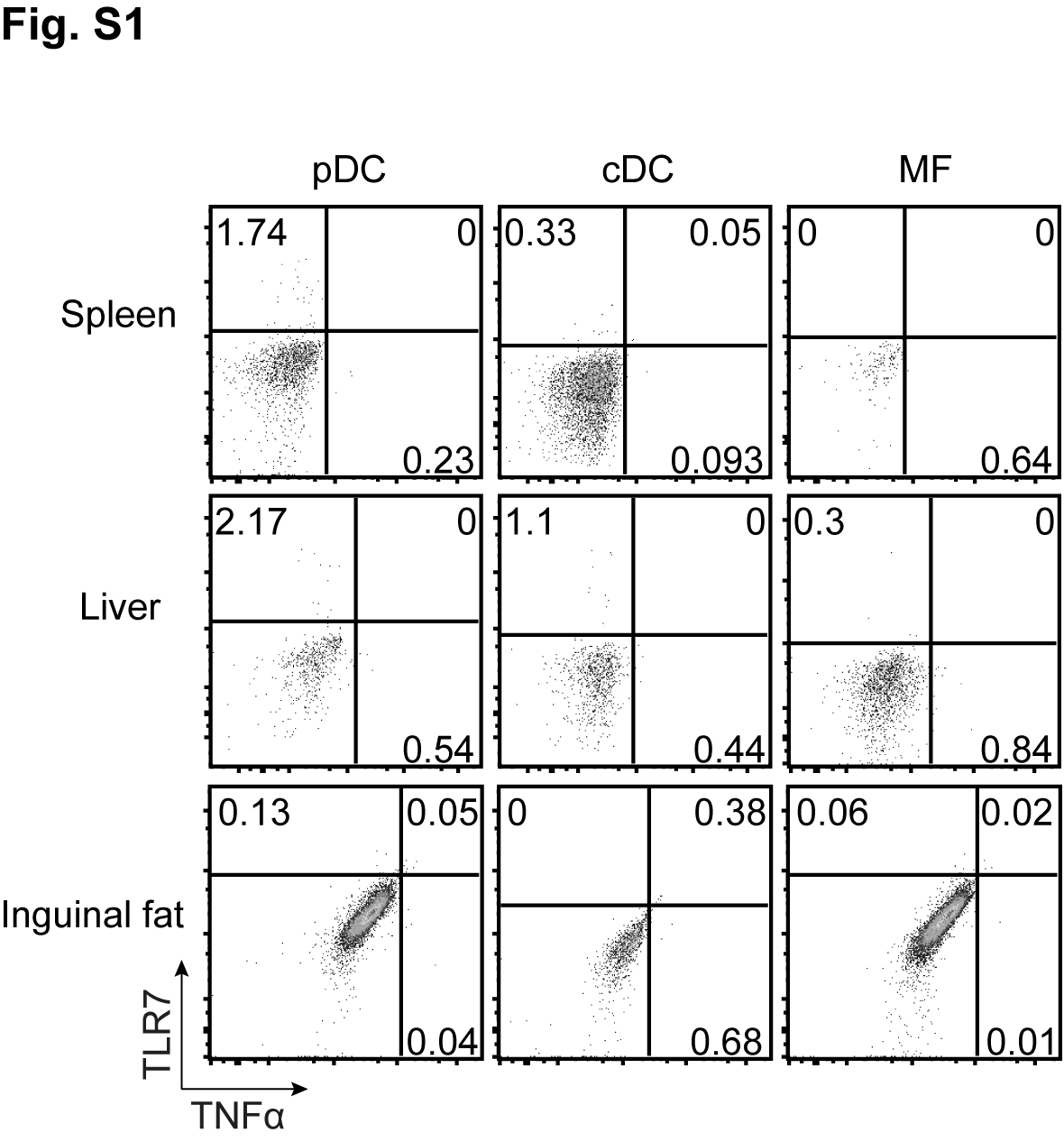

Supplement: Supplementary file 8 [file Image_1.TIF]

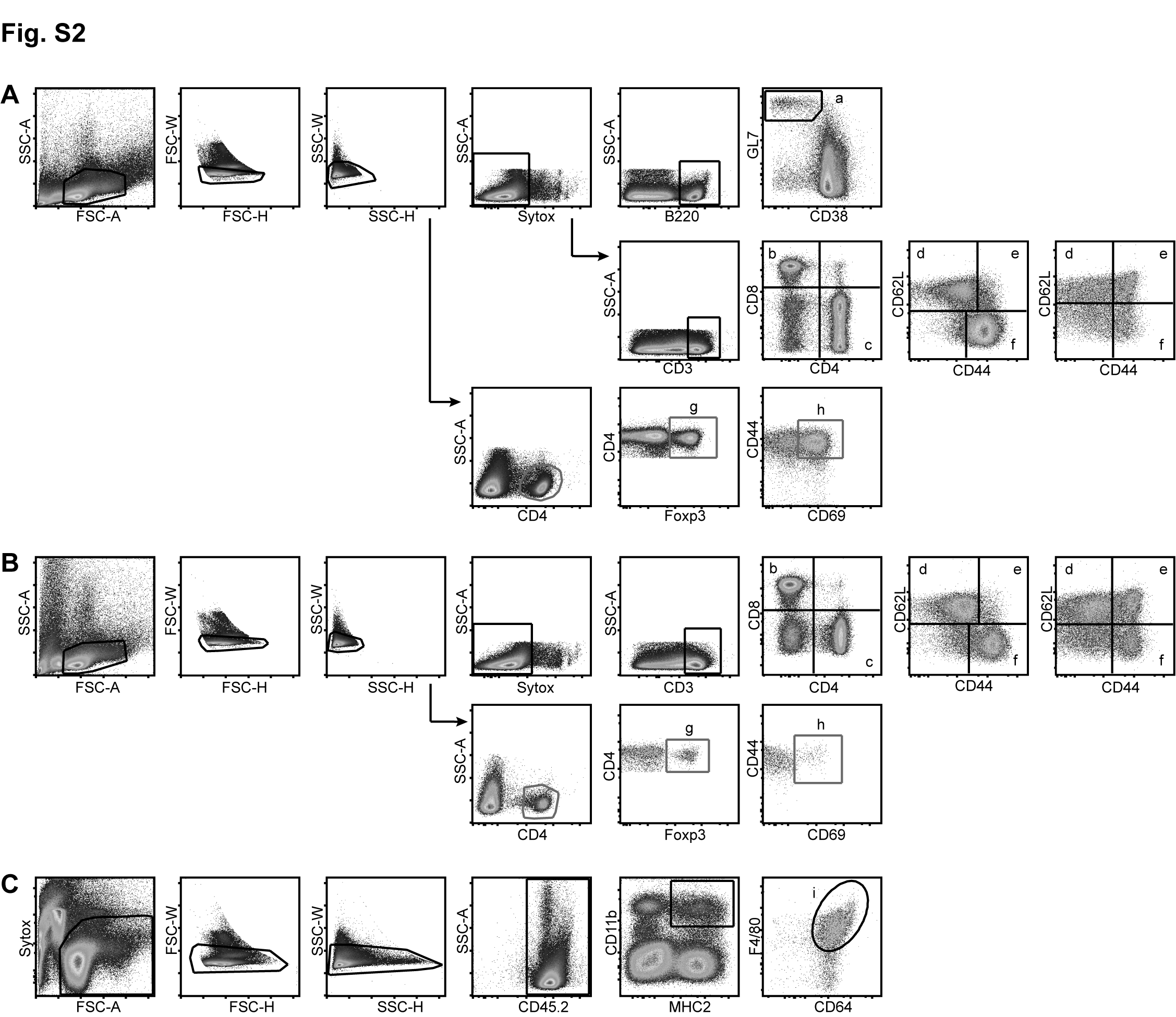

Supplement: Supplementary file 9 [file Image_2.TIF]

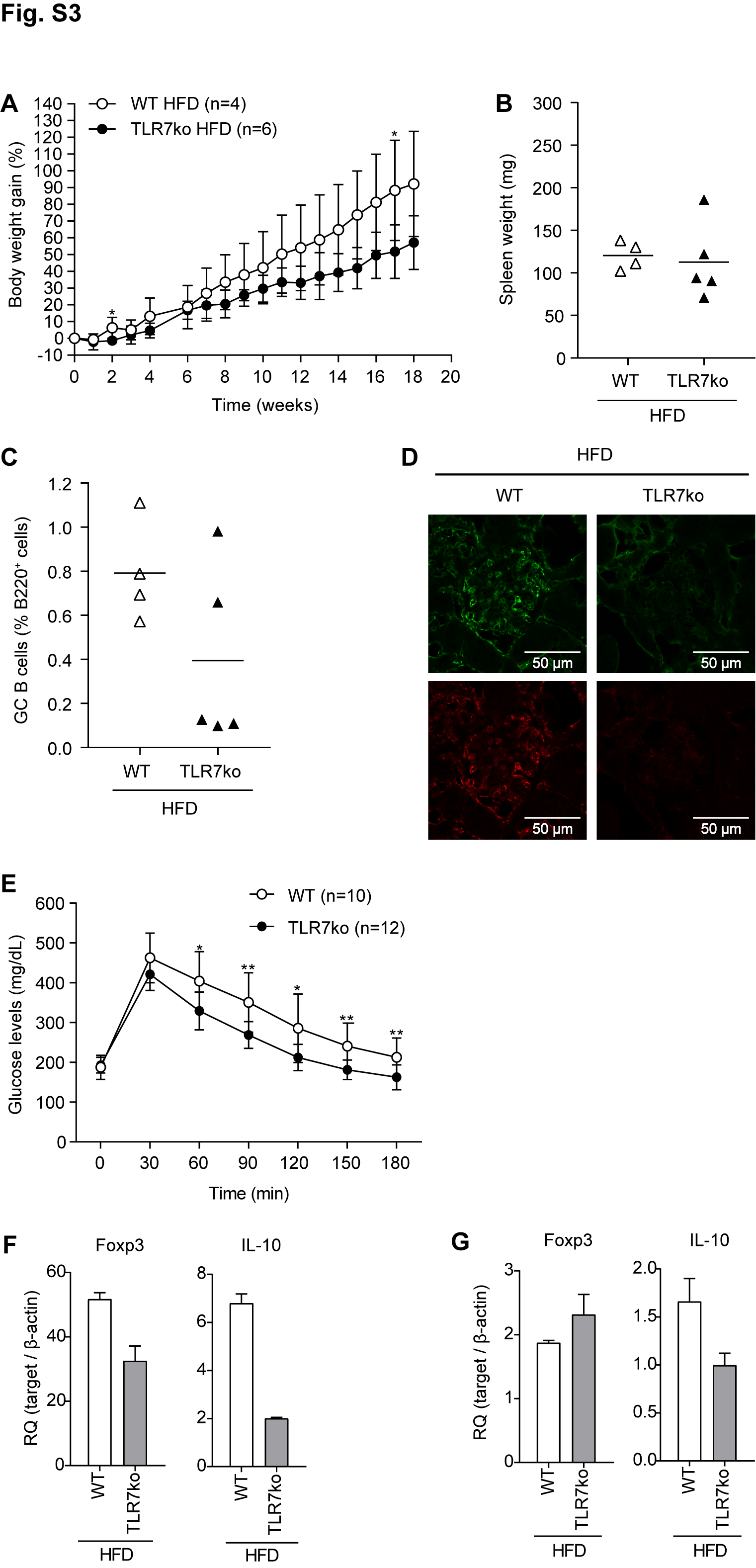

Supplement: Supplementary file 10 [file Image_3.TIF]

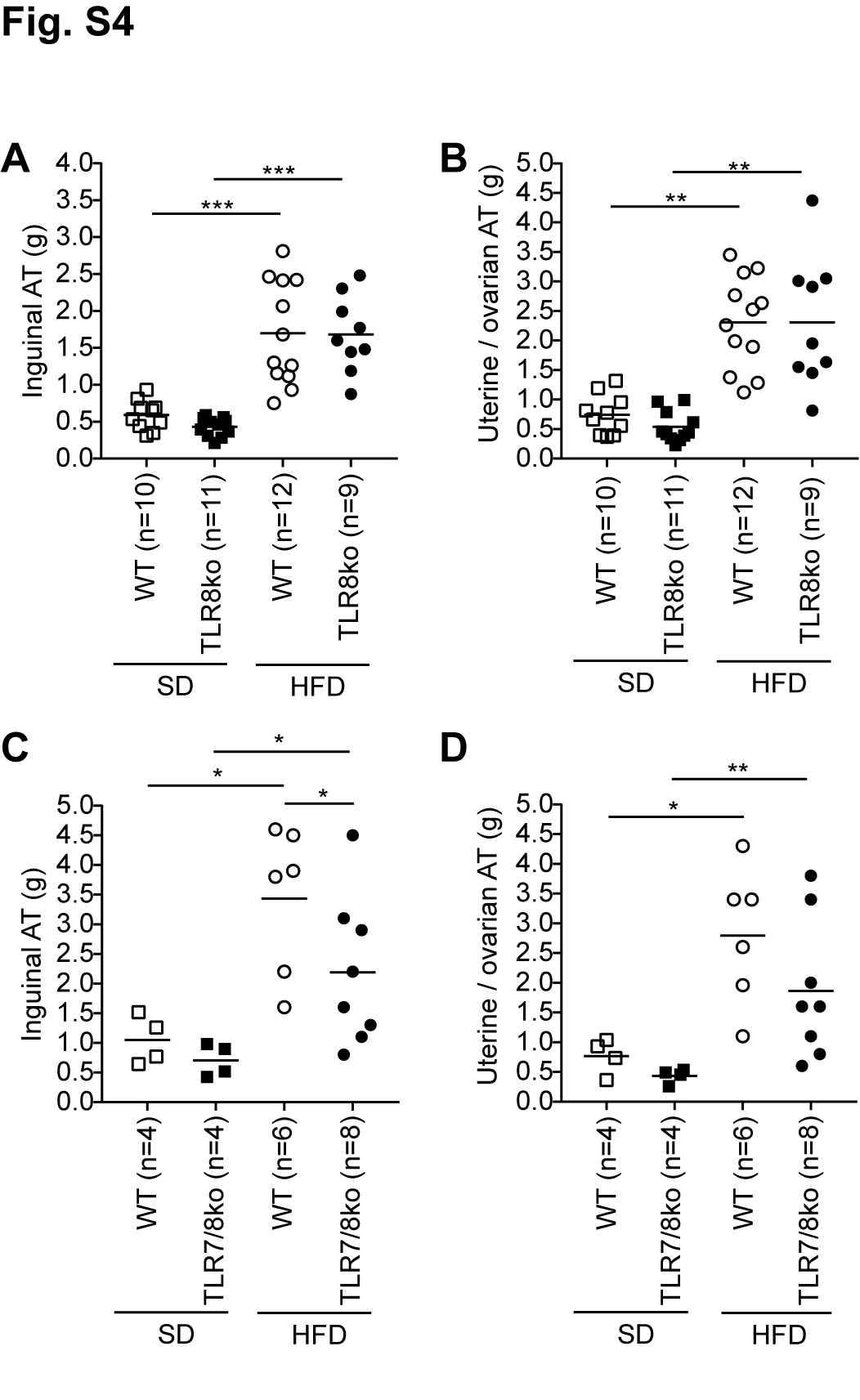

Supplement: Supplementary file 11 [file Image_4.TIF]

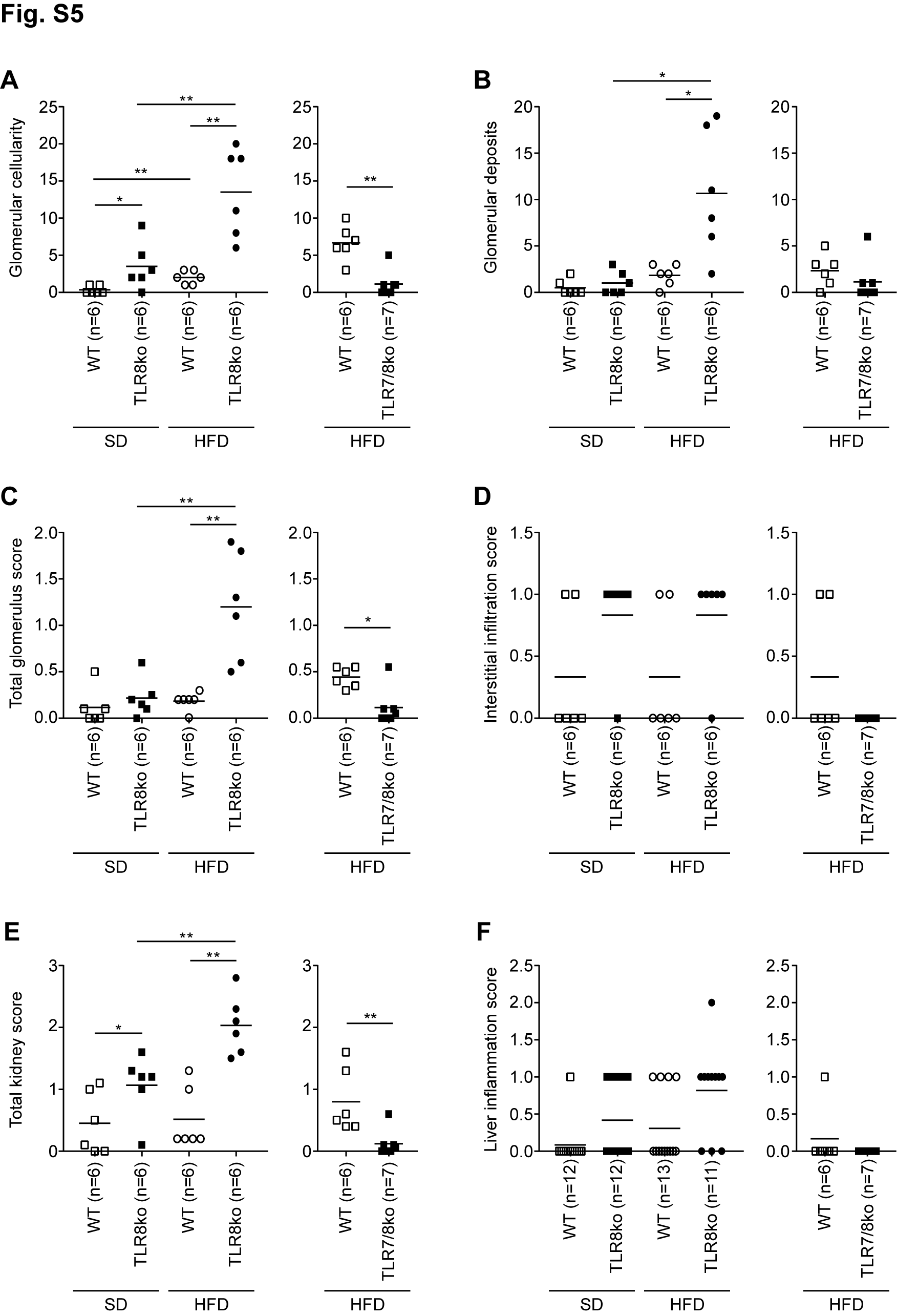

Supplement: Supplementary file 12 [file Image_5.TIFF]
